# A 3D Atlas of Visceral and Somatic Pelvic Motor Neurons in Whole Mounts of Female and Male Rat Spinal Cords

**DOI:** 10.1002/cne.70109

**Published:** 2025-11-16

**Authors:** John‐Paul Fuller‐Jackson, Ziying (Alicia) Yang, Nicole M. Wiedmann, Alan Watson, Nathaniel E. C. Jenkins, Janet R. Keast, Peregrine B. Osborne

**Affiliations:** ^1^ Department of Anatomy and Physiology University of Melbourne Parkville Victoria Australia; ^2^ Center for Biologic Imaging and Department of Cell Biology University of Pittsburgh Pittsburgh Pennsylvania USA

## Abstract

The sexually dimorphic genitourinary organs and specialized striated muscles in the pelvis are controlled by a spinal cord motor neuron (MN) subsystem that produces reflexogenic and psychogenic (conscious) visceral and somatic motor activity during urinary continence and voiding, scent marking (in some species), defecation, reproduction, and sexual activity. To produce a three‐dimensional (3D) atlas of the pelvic MN system, we performed whole‐mount immunostaining and advanced 3D microscopy on the caudal spinal cord of adult female and male rats. We used choline acetyltransferase immunolabeling to study the macroscopic topology of visceral (autonomic preganglionic; VMN) and somatic MN (SMN) subcolumns and retrograde neural tracing from major pelvic ganglia to locate preganglionic neurons required for pelvic organ regulation. This identified parasympathetic VMNs in the sacral intermediolateral nucleus of segments L6–S1 and pelvic sympathetic preganglionic neurons that were mostly found in the medial dorsal commissural nucleus of segments L1–L2. Retrogradely labeled SMNs were also identified in lumbosacral nuclei containing the urinary rhabdosphincter, cremaster, and levator ani motor pools, suggesting that pelvic MNs project through both the pelvic and pudendal nerves. Evidence of sexual dimorphism was provided by VMN counts and measurements of their dendritic arborizations, and the volume of the dorsolateral and dorsomedial SMN columns. Three‐dimensional visualization also revealed areas of overlap between pelvic VMNs and rhabdosphincter SMNs in dendritic bundles, suggesting a possible functional role in coordinating urinary activity. The datasets are available as an open resource (sparc.science) to support 3D visualization of the pelvic motor system in the intact rat spinal cord.

## Graphical abstract

Visceral and somatic motor neurons (MNs) of the lumbosacral (L6–S1) spinal cord control the genitourinary organs and pelvic striated muscles. We produced a three‐dimensional (3D) MN atlas of the lumbosacral spinal cord using whole‐mount immunostaining, tissue clearing, and advanced 3D microscopy in male and female rats. In so doing, we identified novel axon projections and sexually dimorphic features of MN populations, in addition to providing a definitive online open‐access resource for 3D visualization of visceral and somatic MNs within intact lumbosacral cord.

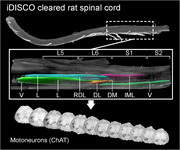

## 1 Introduction

The pelvic motor subsystem, located in the lumbosacral spinal cord, controls the sexually dimorphic urogenital organs and specialized muscles of the pelvis during excretory, sexual, and reproductive behaviors. It produces complex, stereotypic rhythmic motor patterns—such as micturition, defecation, and ejaculation—that require a high degree of cooperativity between the *visceral* motor neurons (VMNs) controlling pelvic organs and the *somatic* motor neurons (SMNs) controlling specialized striated muscles in the pelvic floor and urogenital region (Holstege and Collewijn 2009; Thor and de Groat 2010; Beckel and Holstege 2011; de Groat 2018). The activity of the pelvic motor neurons (MNs) is driven by a series of spinal cord motor pattern generators under reflexogenic control by spinal and brainstem circuits (Steuer and Guertin 2019). However, motor patterns such as voiding during micturition and scent marking are also subject to psychogenic (conscious) controls by brain circuits that integrate these outputs with social and other behaviors (Fowler et al. 2008; Hou et al. 2016; Keller et al. 2018).

The MN system in the spinal cord has been intensively studied as a model for understanding the development and maturation of neural circuits (Blum and Gitler 2022; Dasen 2022). It is formed by the two fundamental neuron classes: SMNs that target striated muscle and VMNs that target peripheral ganglia. Spinal VMNs are also known as autonomic preganglionic neurons, as they provide input to the peripheral autonomic ganglion MNs that control the visceral organs, cardiovascular system, and other motor targets that are not striated muscle. The MNs in the adult spinal cord are hierarchically organized into longitudinal columns—with SMNs extending the length of the spinal cord and VMNs forming noncontiguous columns interrupted by the cervical and lumbar enlargements (Barber et al. 1984). Each column controls a major body segment via MN pools that output to a common functional unit. This organization supports spinal cord motor subsystems that control and coordinate patterned activity in functional groupings of striated muscles or autonomic motor targets.

In rodents, two groups of pelvic VMNs (separated by the lumbar enlargement) are identified by retrograde neural tracing from their primary output target: the bilateral major pelvic ganglia (MPG) (Hancock and Peveto 1979a, 1979b; Hosoya et al. 1994; Harji et al. 1998). The larger caudal group located in the sacral intermediolateral nucleus (IML) in the L6–S1 transition area is classified as parasympathetic VMNs (Anderson et al. 2009; Beckel and Holstege 2011; de Groat et al. 2015; Jobling 2021) that output to the MPG via the pelvic nerve (Nadelhaft and Booth 1984). The smaller rostral group is mostly located in the midline dorsal commissural nucleus (DCN) of segments L1–L2 and is classified as autonomic sympathetic preganglionic neurons that output to the MPG via the hypogastric nerve (Nadelhaft and McKenna 1987). Neural tracing in rodents has also been used to identify the pools of pelvic SMNs targeting the pelvic striated muscles, for example, the urethral rhabdosphincter that controls urine flow (Schrøder 1980; Thor and de Groat 2010; Karnup et al. 2024). Pelvic SMNs are somatotopically organized into motor pools within the lumbosacral transition region, but the male cremaster muscle SMN group is located rostrally in the L1–L2 segments (Kojima et al. 1983). Pelvic SMNs are found in all three major columns specified during development (lateral, medial, and hypaxial) (Dasen 2022), and some motor pools are split across columns (Schrøder 1980; McKenna and Nadelhaft 1986). Many of these motoneurons, for example, those innervating the perineal muscles, are androgen sensitive and sexually dimorphic (Breedlove and Arnold 1980, 1980, 1983a, 1983b; Jordan et al. 1982; Tobin and Payne 1991; Sengelaub and Forger 2008).

The three‐dimensional (3D) organization of the sexually dimorphic pelvic motor subsystem has been extensively studied in multiple species. However, 3D reconstructions have been mostly produced using an ordered series of histological sections that are not contiguous. Here, we have used whole mounts of the lower spinal cord in combination with advanced whole‐mount immunofluorescence and 3D microscopy (Belle et al. 2017; Fuller‐Jackson et al. 2021; Blain et al. 2023) to provide a new perspective of the complex macroscopic neuroanatomical organization of pelvic MNs in female and male rats. We have also performed retrograde tracing from the MPG to specifically visualize spinal neural pathways related to pelvic organ function. To address the limitations of viewing 3D data in 2D figures or videos, the 3D image datasets are shared as a resource available on an open science platform (sparc.science).

## 2 Materials and Methods

### 2.1 Animals

This study used 14 male (300–350 g) and 10 female (200–250 g) adult Sprague–Dawley rats (8–10 weeks of age). The estrus cycle stage of female rats was not recorded. All animal procedures were approved by the Animal Ethics Committee of the University of Melbourne and complied with the Australian Code for the Care and Use of Animals for Scientific Purposes (National Health and Medical Research Council of Australia). Rats were housed in groups of two or more of the same sex under a 12/12‐h light/dark cycle with ad libitum food and water.

### 2.2 Injection of Neural Tracer Into MPG

Pelvic visceral (autonomic preganglionic) MNs in the spinal cord were identified by injecting a retrograde neural tracer, cholera toxin subunit B (CTB), into the paired MPGs located on the surface of the dorsolateral lobe of the prostate (male) or uterine cervix (female). Six rats (three male and three female) were anesthetized with isoflurane (3% in oxygen for induction, 1%–2% for maintenance), followed by a midline ventral incision to access the MPGs. Each MPG was separated from the underlying tissue by blunt dissection with fine angled forceps, and a sterile 2‐mm square piece of parafilm was inserted underneath to minimize leakage of tracer. A solution of CTB (low salt, 0.3% w/v; List Biological Labs, CA, USA) and Evans Blue (0.05% w/v; Sigma–Aldrich, NSW, Australia) in sterile water was then microinjected into the MPG using a glass pipette attached to a Picospritzer (Parker Hannifin). The total volume injected across three microinjections was 1.8–2.2 µL. After injection, the pipette was held in place for 5 s and then withdrawn, and the site was washed with sterile saline.

Following microinjection of both MPGs, the abdominal wall was sutured closed, and the skin was closed with surgical staples. Postoperative analgesia included buprenorphine (Clifford Hallam Healthcare, VIC, Australia; 0.05 mg/kg, at the time of surgery and 8 h postsurgery) and meloxicam (Troy Laboratories, NSW, Australia; 1 mg/kg, 8 and 24 h postsurgery), administered subcutaneously. After surgery, no adverse events were observed. Three days after surgery, tissues were collected (Llewellyn‐Smith et al. 2005).

### 2.3 Tissue Collection

Rats were anesthetized (ketamine 100 mg/kg and xylazine 10 mg/kg, i.p.) prior to tissue fixation via intracardiac infusion, as detailed previously (Keast et al. 2020). Briefly, rats were perfused with 0.9% saline containing 1% sodium nitrite and 5000 IU/mL heparin for 3 min, then 4% paraformaldehyde in 0.1 M phosphate buffer (pH 7.4) for 10 min. The spinal cord was removed and postfixed for 1 h, followed by three 1‐h washes in 0.1 M phosphate‐buffered saline (PBS, pH 7.2). Tissue was stored in PBS with 0.1% sodium azide at 4°C. In one female rat from the CTB injection study, the upper lumbar cord was damaged at dissection and was therefore not analyzed further.

### 2.4 Immunolabeling and Tissue Clearing of Whole‐Mount Spinal Cord

Fixed spinal cords from four female and seven male rats were sub‐dissected to isolate thoracolumbar segments T13–L3 and lumbosacral segments L4–S3. A longer sample of spinal cord (spinal segments T3–S3) was also prepared from one male rat. Segment boundaries were identified by determining the position of the most caudal of the emerging rootlets of each ventral root (CWatson and Kayalioglu 2009; C. Watson et al. 2009). This fiduciary marker could also be identified by light sheet microscopy from the raw signal and was used to identify segments in 3D spinal cord images.

Large‐volume clearing and immunolabeling were performed as previously described (Fuller‐Jackson et al. 2021), using an iDISCO‐based workflow (Renier et al. 2014; Belle et al. 2017). Spinal cords were washed in 1× Dulbecco's PBS (DPBS; Sigma–Aldrich, NSW, Australia; 6 × 15 min), dehydrated in progressively increasing concentrations of methanol (50%, 80%, and 100% in DPBS; 1.5 h each), incubated overnight in 6% hydrogen peroxide in methanol at 4°C, and then rehydrated in decreasing concentrations of methanol (100%, 100%, 80%, and 50%; 1.5 h each). After incubation in DPBS (1.5 h), spinal cords were transferred to a blocking solution for 36 h at room temperature (DPBSG‐T: DPBS with 0.2% gelatin, 0.5% Triton X‐100, and 0.01% thimerosal). Spinal cords were incubated with primary antibodies in DPBSG‐T with an additional 0.1% saponin for 10 days at 37°C, washed (DPBS‐T; DPBS with 0.5% Triton X‐100; 6 × 15 min), and then incubated with secondary antibodies in DPBSG‐T with 0.1% saponin for 4 days at 37°C. Continuing at room temperature, spinal cords were rinsed in DPSB‐T (6 × 15 min), followed by dehydration in methanol in DPBS (20%, 40%, 60%, 80%, and 2 × 100%; 1 h) and incubation in 66% dichloromethane and 33% methanol overnight. Finally, spinal cords were washed three times with 100% dichloromethane for 30 min, optically cleared with dibenzyl ether, and then stored in fresh dibenzyl ether.

Details of primary and secondary antibodies are provided in Table 1. All spinal cords were immunolabeled for choline acetyltransferase (ChAT), with the exception of one spinal cord that was immunolabeled with the neuronal nuclear antigen, NeuN (also known as Fox‐3). Spinal cords from the CTB microinjection study were immunolabeled for CTB and ChAT.

**TABLE 1:** Antibodies used for immunofluorescence microscopy.

Primary antibodies					
RRID	Antigen^a^	Host	Dilution	Source	Catalogue number
AB_11214092	ChAT	Goat	1:500	Millipore	AB144P
AB_258833	CTB	Rabbit	1:30,000 (sections); 1:3000 (iDISCO)	Sigma–Aldrich	C3062
AB_2298772	NeuN	Mouse	1:2000	Chemicon	MAB377

Secondary antibodies						
RRID	Antigen	Tag	Host	Dilution	Supplier	Catalogue number
AB_2307443	Rabbit IgG	Cy3	Donkey	1:3000	Jackson ImmunoResearch	711‐165‐152
AB_2340813	Mouse IgG	Cy3	Donkey	1:2000	Jackson ImmunoResearch	715‐165‐150
AB_10374882	Sheep IgG	AF647	Donkey	1:500	Molecular Probes	A‐21448

^a^ ChAT (choline acetyltransferase), CTB (cholera toxin, B subunit), and NeuN (also known as Fox3).

### 2.5 Large Volume 3D Microscopy

Cleared spinal cords were transferred to ethyl cinnamate and visualized via light sheet microscopy (Ultramicroscope II, Miltenyi Biotec, Germany), using either a zoomable 2x lens (MVPLAPO, Olympus, Japan) or a 12x fixed zoom lens (LVMI PLAN, LaVision, MI, USA). Single‐sided three‐sheet illumination was used with lasers 561 and 639 nm paired with emission filters 620/60 and 680/30 nm. The numerical aperture of the light sheet was set to 0.156. Images were acquired with 2‐µm *z*‐steps and 50–200 ms exposure. The image acquisition area was reduced by ∼25% to minimize the impact of the light sheet waist on the uniformity of the image during mosaic acquisitions, which were performed with 10% overlap between image stacks. Image stacks were converted to Imaris file types (Imaris File Converter, Bitplane, UK) and stitched using Imaris Stitcher, prior to detailed visualization in Imaris (RRID:SCR_007370).

ChAT immunofluorescence in two cleared spinal cord whole mounts was also visualized using ribbon scanning confocal microscopy (A. M. Watson et al. 2017). The microscope (Caliber, MA, USA) was fitted with a Nikon CFI90 20x glycerol‐immersion objective (Nikon, NY, USA) with 8.3 mm working distance. Volumes were captured with voxel resolution of 0.491 × 0.491 × 10.67 µm (*x*, *y*, *z*). Laser intensity and detector settings were specific to each sample based on the levels of staining. In all cases, the intensity of the laser was increased by linear interpolation throughout deeper focal planes to compensate for absorption of excitation and emission light. Images acquired in this way were stitched and assembled into composites using a 24‐node, 608‐core cluster, then converted into the Imaris file format. Volumes were rendered using Imaris.

Three‐dimensional representation of motor nuclei was achieved using the *Surfaces* function in Imaris, with contours drawn manually around motor nuclei in either transverse, sagittal, or horizontal optical slices. Motor nuclei were identified according to their ChAT^+^ cytoarchitecture and MN morphology (Schrøder 1980; C. Watson et al. 2009, 2021).

### 2.6 Quantitation of Spinal Neurons in Specific Nuclei

VMNs and SMNs were manually counted in raw images from cleared spinal cord whole mounts by using Neurolucida 360 (MBF Bioscience, VT, USA, RRID:SCR_016788) in the 2D view to mark single neurons that were in regions identified in horizontal 2‐µm‐thick virtual slices. All the VMNs were counted by one individual, while a separate individual counted the SMNs. Pelvic VMNs were identified by the appearance of CTB in their somata as a result of retrograde neural tracer injections into the MPGs. In L6–S1 segments, ChAT^+^/CTB^+^ and ChAT^+^/CTB^−^ neurons were counted in the IML on one side of the cord, and the ratio of counts was used to calculate the efficiency of retrograde labeling. In the L1–L2 segments, the neuron classes were counted in the principal IML on one side and in the midline DCN, but retrograde labeling efficiency was not estimated, as these regions do not exclusively target the MPG and contain other classes of nonpelvic VMNs. We also identified CTB^+^ SMNs that were manually counted.

### 2.7 Morphological Analysis of Motor Nuclei That Include Anal and Urethral Rhabdosphincter MNs

The dorsomedial and dorsolateral motor columns were analyzed in six rats of each sex. These sections were immunolabeled for a previous study (Fuller‐Jackson et al. 2021); however, in the current study, this open dataset was reanalyzed to quantify the area of each motor nucleus in each section. In this previous study, lumbosacral spinal cords (L5–S2) were cryoprotected overnight in 0.1 M PBS containing 30% sucrose prior to embedding in Tissue‐Tek optimal cutting temperature compound (Sakura Finetek, CA, USA). Transverse cryosections (40 µm) of the entire block were collected in order, with alternate sections immunolabeled for ChAT. Free‐floating sections were washed in 0.1 M PBS, blocked in 0.1 M PBS with 10% normal horse serum (NHS) and 0.5% Triton X‐100 for 2 h, washed in 0.1 M PBS, and then incubated in a solution containing primary antibodies, 2% NHS, 0.5% Triton X‐100, and 0.1% sodium azide for 48 h at room temperature. After washing in 0.1 M PBS, sections were incubated in species‐specific secondary antibodies tagged with AF647 in a solution of 0.1 M PBS containing 2% NHS and 0.5% Triton X‐100 for 4 h at room temperature. Mounted on glass slides in rostrocaudal anatomic order, sections were cover‐slipped using carbonate‐buffered glycerol (pH 8.6). Primary and secondary antibodies are described in Table 1.

All spinal cord sections were imaged (10x objective, pixel scaling 0.645 × 0.645 µm, five *z*‐steps at 5 µm) with a Zeiss AxioImager Z1 (Carl Zeiss Microscopy). Sections were aligned in TissueMaker (MBF Bioscience, PA, USA; RRID:SCR_017322), producing a reconstructed spinal cord dataset (Fuller‐Jackson et al. 2021). The distance between sections was 80 µm. In Neurolucida 360, dorsolateral and dorsomedial motor columns were identified, and the outer boundary of each nucleus was manually traced in each section using the *Contour* function. This involved tracing around the collection of neurons as they appear in the section and was performed bilaterally in each section containing these nuclei. For the dorsomedial column, the entire rostrocaudal extent of this nucleus was mapped. For the dorsolateral column, only sections where the dorsolateral column was the sole nucleus in the ventrolateral region were mapped. This excluded rostral sections that contained both dorsolateral and lateral motor columns in the same region, which were not mapped due to the difficulty in accurately distinguishing the boundaries of each column. In Neurolucida Explorer (MBF Bioscience; RRID:SCR_017348), the area of each contour on each section was determined across all relevant sections. Data were plotted as motor nucleus area (µm^2^) from the most rostral section to the most caudal section. In each animal, the total contour area from each nucleus was then calculated and compared between the male and female groups.

### 2.8 Analysis of Dendritic Fields of Caudal Lumbosacral Visceral (Preganglionic) MNs

The distribution of CTB in parasympathetic preganglionic neurons in the L6–S1 segments was used to visualize the features of their dendritic fields in 3D in cleared spinal cord of male and female rats. In dendrites, CTB granules were punctate in appearance. Using the *Spots* function in Imaris, spherical 3D objects were detected according to their size (1 µm *XY*, 2 µm *Z*) and signal intensity after background subtraction. Due to the punctate nature of CTB within neuronal processes, individual dendrites could not be traced.

To generate the total volume of these dendritic fields, CTB objects in Imaris were filtered prior to the fitting of a convex hull around the remaining objects. Alpha Shapes creates polygons from objects according to a set parameter (α). The higher the α, the greater the distance between objects that can be included in the creation of polygons using *Alpha Shapes*. For this study, α was set to 3–4, based on visual assessment of each dataset. This effectively excluded distant objects that were isolated from other objects. The final step, the fitting of a convex hull, involved the generation of multiple smaller convex hulls along the length of the dataset. Initially, a single convex hull was applied; however, each of the most distant CTB points in any given direction created a convex hull that overestimated the volume of the dendritic field. Smaller convex hulls for subsets of CTB points sorted by *Z* were generated, with 20%–30% overlap of CTB points between each adjacent convex hull. The union of the smaller convex hulls improved the mapping of the contours of dendritic projections. The overlaps between convex hulls were removed, and these convex hulls were union‐fused to produce a single watertight volume.

Our method was developed as a Python pipeline assembled by open‐sourced Python libraries and our own algorithms (https://gitlab.unimelb.edu.au/lab‐keast‐osborne‐release/3d‐points‐volume‐estimation). The Python library, PyMeshlab (Muntoni and Cignoni 2021), was employed to create the alpha shape in the initial step. The filtered spots were then used to compute all the described smaller convex hulls using Trimesh (Dawson‐Haggerty 2019). Finally, the union of the convex hull shapes was processed by PyMeshlab. We also used Numpy (Harris et al. 2020) and Pandas (The Pandas Development Team 2020) in our program.

### 2.9 Tracing Axon Projections From Sacral SMNs

Axons of sacral (S1) SMNs were traced within the spinal cord white matter. These included axons that projected laterally into the white matter tracts, before turning perpendicularly to continue either rostrally or caudally to the next segment. Cleared lumbosacral spinal cord immunolabeled with ChAT was imaged via light sheet microscopy at 12x magnification, and the resulting Imaris files were converted to JPEG2000 using Microfile+ (MBF Bioscience, RRID:SCR_018724). Using Neurolucida 360's *Tree tracing* function in the 3D environment, axons of sacral SMNs were traced by performing user‐guided tracing with the *Directional Kernels* algorithm of choice. Where two traced axons were in close proximity, the Smart‐manual tracing tool was used, thereafter returning to user‐guided tracing. Axons were traced as far as they could be confidently identified as the same axon.

### 2.10 Antibody Characterization

Primary antibodies were obtained from the following sources (information on specificity was obtained from the manufacturer):

ChAT (Millipore; AB144P; batch 2971003; RRID:AB_11214092): immunoaffinity‐purified polyclonal antibody raised in goat against the human placental enzyme. In western blotting, 70‐kDa bands were detected in NIH/3T3 lysate, matching the molecular weight of ChAT protein.
NeuN (Chemicon; MAB377; batch 2453249; RRID:AB_2298772): purified monoclonal antibody raised in mouse against purified cell nuclei from mouse brain. In western blotting, 2–3 bands were recognized in the 46–48 kDa range, matching the molecular weight of NeuN.
CTB (Sigma–Aldrich; C3062; batch 048M4780V; RRID:AB_258833): polyclonal antibody raised in rabbit against Vibrio cholerae, reacting with cholera toxin but not staphylococcal enterotoxin A, staphylococcal enterotoxin B, and pseudomonas exotoxin A.

### 2.11 Experimental Design and Statistical Analysis

For the qualitative description and representation of spinal cord motor nuclei, long (T3–S3) and short (L4–S3) spinal cords from male rats were used. One male and one female rat spinal cord (L5–S2) were used to demonstrate sexually dimorphic motor nuclei in the intact cord, while cryosections from spinal cords of six male and six female rats were used for the quantitative comparison of these motor nuclei. Plots of the total area of motor nuclei contours in sections across the neuraxis of L6 were used to compare the size of the motor nuclei in male and female rats. Spinal cords from three male and three female rats that received MPG microinjections of CTB were used for the quantitative comparison of neuron number and dendritic field volume of sacral preganglionic neurons. Comparisons between sexes were made using a two‐tailed *t‐*test. Graphs were generated in JMP 16.0 (SAS Institute, NC, USA; RRID:SCR_014242), and data are presented as group mean and individual subjects. Statistical analyses were performed using JMP 16.0.

### 2.12 Figure Production

For representative images of light sheet datasets, neurons were visualized with γ set in Imaris to 1.4 (ChAT, CTB) or 1.6 (NeuN). To best demonstrate neuronal morphology in 2D outputs from Imaris, small linear adjustments were made to levels using Photoshop (Adobe Creative Suite v2023). In some figures, the ventral roots have been nondestructively removed from view using Photoshop, particularly when intense ChAT staining of roots distracts from fine neuronal features within the spinal cord image. Figures were constructed in InDesign (Adobe Creative Suite v2023).

### 2.13 Data Sharing

Raw data from this study will be published under an open‐access license on https://sparc.science/. Movies illustrating the complete 3D datasets are also available online (DOI for each Movie provided where relevant in Section 3).

## 3 Results

### 3.1 Visceral (Preganglionic) and Somatic MNs in Caudal Spinal Cord

**FIGURE 1:**
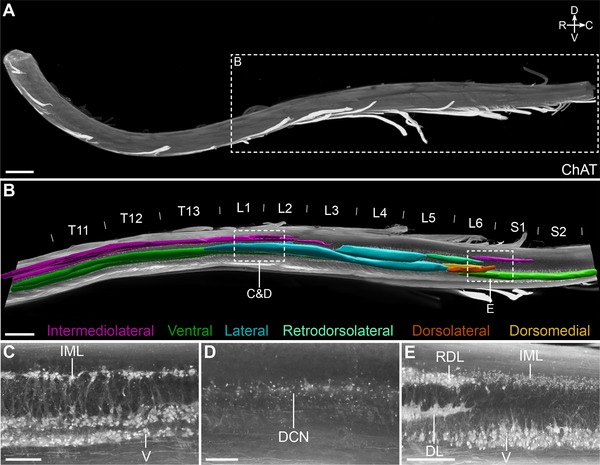
Distribution of motor columns and pools in the caudal male rat spinal cord. (A) Intact, cleared spinal cord of a male rat containing segments T3–S2 immunolabeled with choline acetyltransferase (ChAT) and visualized with ribbon scanning confocal microscopy. (B) Three‐dimensional segmentation of motor pools shown in a sagittal orthogonal projection of the caudal spinal cord. From the same dataset, example orthogonal projections in the sagittal plane of the upper lumbar cord showing (C) the intermediolateral nucleus (IML), (D) the dorsal commissural nucleus (DCN), and (E) the lumbosacral IML. The DCN was unable to be segmented due to its indistinct boundaries and limited capacity to distinguish preganglionic neurons from ChAT+ interneurons. L, lateral; V, ventral; RDL, retrodorsolateral; DL, dorsolateral; DM, dorsomedial (Schrøder 1980). Scale bars: (A) 2000 µm, (B) 1000 µm, and (C–E) 300 µm.

Multichannel ribbon scanning confocal microscopy is a relatively new technology that uses a resonant single scan head coupled to a fast stage to scan continuous “ribbons,” which increases the speed of acquisition and reduces the amount of stitching required to produce tiled image mosaics (A. M. Watson et al. 2017). This supports imaging of very large whole‐mount samples at an equivalent resolution to conventional confocal microscopy. We prepared spinal cord whole mounts from two rats and used ribbon scanning to collect images of the intact lower spinal cord extending from segment S2 to as far rostral as segment T3. The caudal limit of the rootlets in each of the ventral roots was visible in the background fluorescence and provided a reliable fiduciary marker to identify the lower boundary of each segment (C. Watson and Kayalioglu 2009; C. Watson et al. 2009). Using ChAT immunolabeling, we could identify and visualize the VMN (preganglionic) and SMN columns extending from segments T10 to S2 (Figure 1A; Media 1 [https://doi.org/10.26188/25567014.v1]). The macroscopic 3D topology of the MN subcolumns (i.e., Lamina 9 nuclei, Barber et al. 1984) was revealed by segmenting with Imaris software (Figure 1B). This showed how the lateral motor columns extend through most of the lumbar enlargement, spanning the discontinuity in the intermediolateral preganglionic columns, that is, between caudal thoracic (sympathetic) and caudal lumbar (parasympathetic) segments (Figure 1B). This 3D approach also effectively demonstrated the complexity in rostrocaudal organization of individual subcolumns of MNs (Schrøder 1980; Barber et al. 1984) (Table 2), especially between segments L5 and S1, where numerous aggregates of SMNs partially overlap in their segmental distribution and alignment with VMNs. The resolution of ribbon scanning (*XY* resolution of 0.491 µm and with optical slices spaced 10.7 µm apart in the Z axis) also revealed cellular morphology of all ChAT^+^ MNs and interneurons (Figure 1C–E) in the spinal cord whole mount. As described previously (Barber et al. 1984), ChAT^+^ MNs were most commonly larger multipolar or bipolar neurons that were aggregated into columns or groups of neurons extending longitudinally through the intermediate gray matter or ventral horn (Figure 1C–E). By contrast, ChAT^+^ interneurons were mostly identified as isolated neurons located in intermediate laminae ventral to the central canal as well as in the dorsal horn.

**TABLE 2:** Neuroanatomical classification of pelvic somatic motor neurons (MNs).

Somatic muscle target	Lumbosacral lamina 9 MN nuclei identified by neural tracing (number of studies)	Citations	
		Female	Male
Urethral rhabdosphincter	Dorsolateral n. (8) Lateral n. (1) Ventral n. (1)	McKenna and Nadelhaft 1986 ^SD,F,M^; Koliatsos et al. 1994 ^SD,F,M^; Xu et al. 2007 ^SD,F,M^; Fuller‐Jackson et al. 2021 ^SD,F,M^	Schrøder 1980 ^W,M^; McKenna and Nadelhaft 1986 ^SD,F,M^; Canon et al. 1993 ^W,M^; Koliatsos et al. 1994 ^SD,F,M^; Canon et al. 1991 ^W,M^; Xu et al. 2007 ^SD,F,M^; Nadelhaft and Vera 2001 ^SD,M^; Fuller‐Jackson et al. 2021 ^SD,F,M^
Anal rhabdosphincter	Dorsomedial n. (6) Ventral n. (1)	McKenna and Nadelhaft 1986 ^SD,F,M^; Koliatsos et al. 1994 ^SD,F,M^	Schrøder 1980 ^W,M^; McKenna and Nadelhaft 1986 ^SD,F,M^; Canon et al. 1993 ^W,M^; Koliatsos et al. 1994 ^SD,F,M^; Canon et al. 1991 ^W,M^; Xu et al. 2007 ^SD,M^
Bulbocavernosus	Dorsomedial n. (9)		Schrøder 1980 ^W,M^; McKenna and Nadelhaft 1986 ^SD,M^; Canon et al. 1991 ^W,M^; Canon et al. 1993 ^W,M^; Breedlove and Arnold 1980 ^SD,M^; Collins et al. 1991 ^SD,M^; Peshori et al. 1995 ^SD,M^; Koliatsos et al. 1994 ^SD,M^; Xu et al. 2007 ^SD,M^
Ischiocavernosus	Dorsolateral n. (7) Ventral n. (1)		Schrøder 1980 ^W,M^; McKenna and Nadelhaft 1986 ^SD,M^; Canon et al. 1993 ^W,M^; Canon et al. 1991 ^W,M^; Breedlove and Arnold 1980 ^SD,M^; Koliatsos et al. 1994 ^SD,M^; Xu et al. 2007 ^SD,M^
Levator ani	Ventral n. (4) Dorsomedial n. (1)	Cuevas et al. 2006 ^W,F^	Schrøder 1980 ^W,M^; Canon et al. 1993 ^W,M^; Canon et al. 1991 ^W,M^; Manzo et al. 1999 ^W,M^; Cuevas et al. 2006 ^W,F^; Breedlove and Arnold 1980 ^SD,M^; Dobberfuhl et al. 2014 ^SD,M^

Abbreviations: F, Female; M, Male; SD, Sprague–Dawley; W, Wistar.

### 3.2 VMN and SMN Pools in the Lumbosacral Transition Area

**FIGURE 2:**
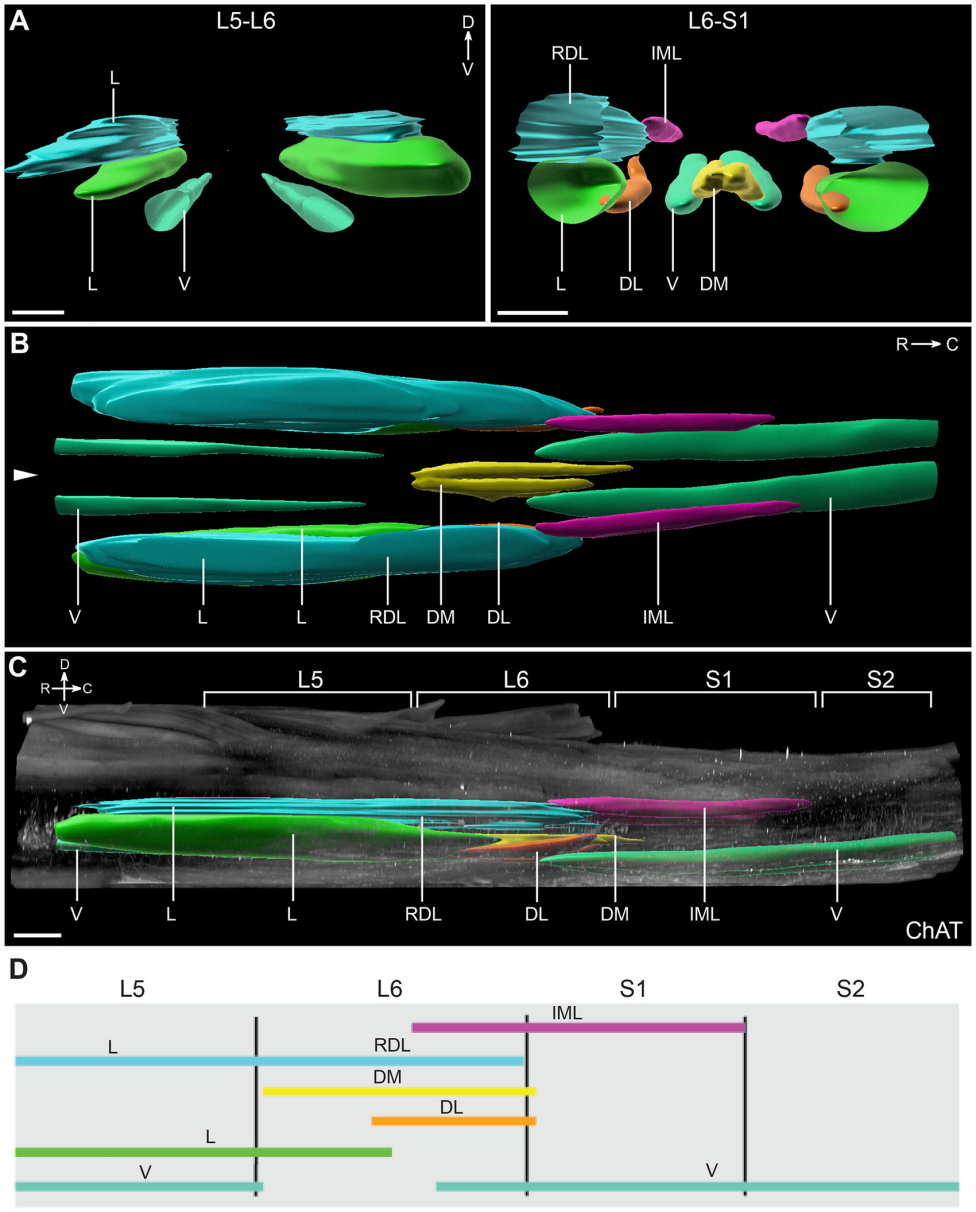
Three‐dimensional mapping of lumbosacral motor pools. (A) Transverse view of isolated segmented motor pools in the rostral (L5–L6) and caudal (L6–S1) lumbosacral spinal cord. (B) Horizontal visualization of the segmented motor pools in the lumbosacral spinal cord. Midline indicated by arrowhead. (C) Orthogonal projection in the sagittal view of the lumbosacral spinal cord (male) with segmented motor pools superimposed on immunolabeling for choline acetyltransferase (ChAT). (D) Schematic of the rostrocaudal distribution of motor and premotor nuclei from L5 to S2. Mapping based on datasets from ribbon scanning confocal microscopy. L, lateral motor column; V, ventral motor column; RDL, retrodorsolateral motor column; DL, dorsolateral motor column; DM, dorsomedial motor column; IML, intermediolateral nucleus; V, ventral motor column. Scale bar: 500 µm (applies to all panels).

The neuroanatomical organization of MNs in the lumbosacral transition area has unique features to support control of sexually dimorphic visceral–somatic motor function. This includes locating pelvic VMNs (parasympathetic preganglionic MNs) close to the SMN pools that target striated muscles relevant to urogenital and lower bowel function (Schrøder 1980). To demonstrate how lumbosacral VMN and SMN columns organize in 3D space, we next used light sheet microscopy to image ChAT^+^ neurons in contiguous L5–S2 segments of cleared spinal cord whole mounts. As shown in Figure 2, the 3D image volumes were then segmented to identify and visualize the sacral IML (frequently identified in previous studies as the sacral preganglionic nucleus, SPN) containing the column parasympathetic preganglionic column of VMNs in sacral IML, and the five columns of SMNs located in the lumbosacral transition area (Schrøder 1980) (Table 2). These annotated image datasets can be rotated and viewed from any perspective but are presented as orthogonal projections from conventional transverse (Figure 2A), horizontal (Figure 2B), and sagittal (Figure 2C) viewpoints.

We used a schematic (Figure 2D) to summarize the complex rostrocaudal positioning and overlap of MN columns in the lumbosacral spinal cord. This shows how in S1, the sacral IML containing the VMN column only overlaps with the ventral SMN columns but then extends into the lower half of L6, where it overlaps with four SMN columns (dorsolateral, dorsomedial, retrodorsolateral, ventral). It is then absent from the rostral half of L6. This complex 3D topology of MN columns within these segments aligns with previous identification of a transition zone between rostral SMN pools in the lumbar enlargement and caudal VMNs in the sacral preganglionic column (sacral IML) (Schrøder 1980).

### 3.3 Retrograde Tracing Identifies Pelvic VMNs and Reveals Novel Somatic Motor Pathways Projecting via the MPG

**FIGURE 3:**
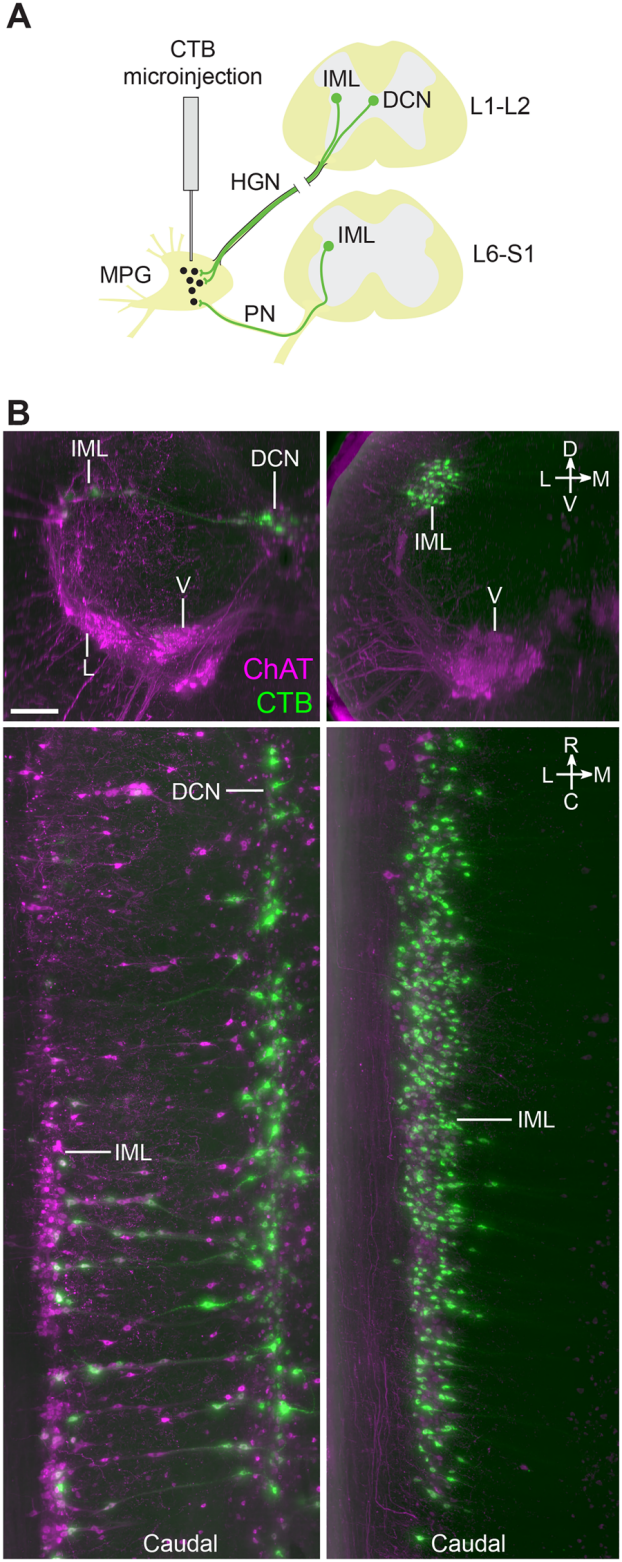
Retrograde labeling of preganglionic neurons in the lumbosacral spinal cord that project to the major pelvic ganglia. (A) Schematic representation of cholera toxin subunit B (CTB) microinjection into the rat major pelvic ganglion with resultant retrograde labeling in preganglionic neuron populations in the spinal cord. (B) Virtual slices of CTB‐filled preganglionic neurons in cleared L1–L2 (left) and L6–S1 (right) spinal cord (male). Spinal cord immunolabeled for CTB and choline acetyltransferase (ChAT). The top panels show the spinal cord in transverse orientation, whereas the lower panels show the columns of preganglionic neurons in the rostrocaudal axis. Images are oriented with the midline to the right. HGN, hypogastric nerve; PN, pelvic nerve; IML, intermediolateral nucleus; DCN, dorsal commissural nucleus; L, lateral motor column; V, ventral motor column. Scale bar: 200 µm (applies to all panels).

Spinal VMNs controlling pelvic organ function are split into two functionally distinct groups of sympathetic and parasympathetic preganglionic neurons, which in rats are, respectively, located on either side of the lumbar enlargement in the L1–L2 and L6–S1 segments of the cord. SMNs that innervate striated muscle related to urogenital or lower bowel function (urethral and anal sphincters, pelvic floor) are also located in the caudal lumbosacral segments, except for a smaller rostral group in segments L1–L2 that target the cremaster muscle. To visualize the 3D structure and location of both sympathetic and parasympathetic pelvic preganglionic neurons, we injected the MPG of three male and three female rats with the retrograde neural tracer CTB (Figure 3A). In rodents, this ganglion contains most of the autonomic neurons that innervate tissues of pelvic organs and is the site where both sympathetic and parasympathetic preganglionic neurons synapse. These preganglionic axons project via the hypogastric and pelvic nerves (Figure 3A). Spinal neurons labeled by CTB injection into the MPG are also shown in Media 2 (https://doi.org/10.26188/25479250.v1) and Media 3 (https://doi.org/10.26188/25484116.v1). In the upper lumbar region of the spinal cord, CTB^+^/ChAT^+^ preganglionic neurons were restricted to neuronal columns in segments L1–L2 (Figure 3B; Media 3 [https://doi.org/10.26188/25484116.v1]) and were more densely distributed in the midline DCN than in the lateral IML. In the lumbosacral IML (L6–S1), the source of parasympathetic pelvic preganglionic neurons, most of these ChAT^+^ neurons were labeled by CTB (Figure 3B; Media 2 [https://doi.org/10.26188/25479250.v1]), indicating the effectiveness of our retrograde tracing method. A minority of SMNs in specific locations were also labeled (discussed below).

We then manually counted CTB^+^ neurons in the preganglionic nuclei, analyzing the IML on one side and the entire DCN (Table 4). In the L6–S1 IML (the sole location of parasympathetic preganglionic neurons), we also counted the entire population of ChAT^+^ neurons to provide an estimate of our CTB labeling efficiency. This indicated that 85.0 ± 3.3% (mean ± SEM, *n* = 6, range 71%–93%) of lumbosacral preganglionic neurons were CTB^+^. Our analysis of the total preganglionic neuron population on one side of L6–S1 revealed a sex difference (male > females; males, 844 ± 30, *n* = 3 vs. females, 707 ± 31, *n* = 3; two‐tailed *t‐*test, *p* = 0.033, *df* = 5; Table 4).

Similar quantitation of CTB^+^ preganglionic neurons in the L1–L2 segments from the same animals (Table 4) showed that fewer neurons were labeled with CTB than in the L6–S1 segments (L1–L2, 137–369; L6–S1, 589–723). The majority (65%–89%) of the CTB^+^ preganglionic neurons in L1–L2 were in the DCN. In the L1–L2 IML, CTB^+^ neurons comprised only 4%–11% of all preganglionic neurons identified by ChAT immunoreactivity. Sex differences in these segments could not be analyzed due to the smaller number of biological replicates.

**FIGURE 4:**
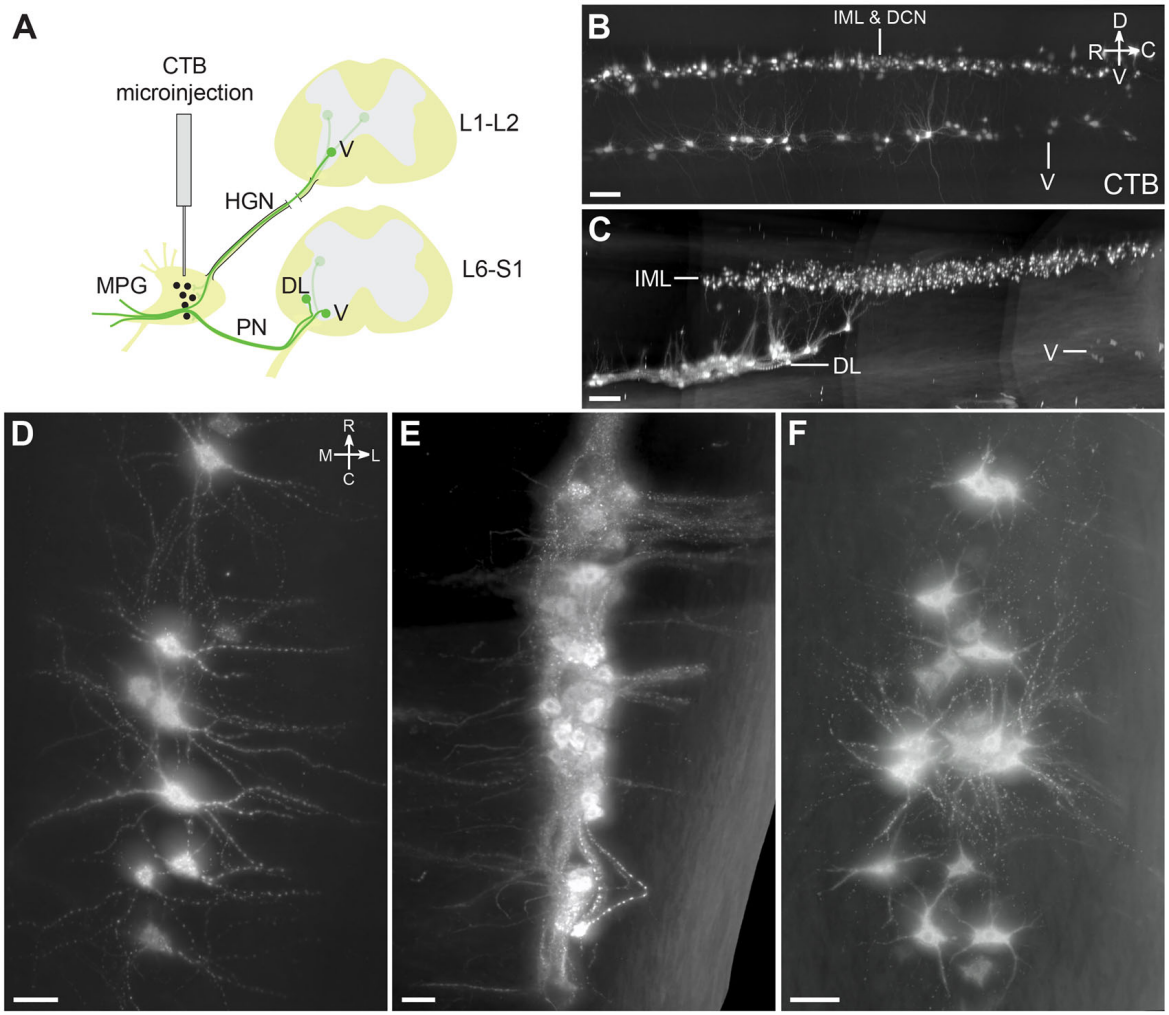
Somatomotor projections via the major pelvic ganglion and visceral nerves, revealed by cholera toxin subunit B retrograde tracing (CTB). (A) Schematic representation of CTB microinjection into the rat major pelvic ganglion with resulting retrograde labeling in motor neuron populations in the lumbosacral spinal cord. The inferred route of this trajectory via the hypogastric and pelvic nerves is indicated. Sagittal view of CTB‐positive motor neurons in (B) L1–L2 and (C) L6–S1 cleared spinal cord (male). Horizontal virtual slices of CTB‐positive motor neurons in (D) cremaster (ventral, V), (E) urethral rhabdosphincter and ischiocavernosus (dorsolateral, DL), and (F) levator ani and tail (ventral, V) motor nuclei. B–E, male; F, female. HGN, hypogastric nerve; PN, pelvic nerve; IML, intermediolateral nucleus; DCN, dorsal commissural nucleus. Scale bars: (B, C) 200 µm and (D–F) 50 µm.

CTB injected into the MPG did not label ChAT^+^ interneurons or ChAT^−^ neurons in any spinal segment. However, unexpectedly, a subpopulation of ChAT^+^ SMNs in specific nuclei was labeled by CTB (Figure 4A; Table 4). These neurons had large, rounded somata with the multipolar morphology characteristic of alpha SMNs, and few or none had the spindle‐shaped morphology of the smaller gamma SMNs (Friese et al. 2009; Burke 2016). In both male and female rats, CTB labeled S1 SMNs in the ventral motor column (Figure 4B,F; Media 2 [https://doi.org/10.26188/25479250.v1]). Neural tracing has shown that the primary targets of lumbosacral ventral SMNs include the levator ani muscle in the pelvic floor and the tail muscles. However, a comparison of three male rats and two female rats also identified a sex difference (Table 4). In male rats, but not female rats, CTB labeled a rostral group of ChAT^+^ SMNs in the L1–L2 ventral motor column and a caudal group in the L6 dorsolateral motor columns. Using atlases of the rat spinal cord (C. Watson et al. 2009, 2021), the group of ventral CTB^+^ SMNs in L1–L2 (Figure 4B,D; Media 3 [https://doi.org/10.26188/25484116.v1]; Table 4) were mapped to the nucleus containing the cremaster SMNs that supply the involuntary striated muscle responsible for retracting the testicles, and the group of dorsolateral CTB^+^ SMNs in L6 (Figure 4C,E; Media 2 [https://doi.org/10.26188/25479250.v1]; Table 4) were localized to the nucleus containing urethral rhabdosphincter SMNs.

### 3.4 Comparison With 2D Rat Spinal Cord Atlas

**FIGURE 5:**
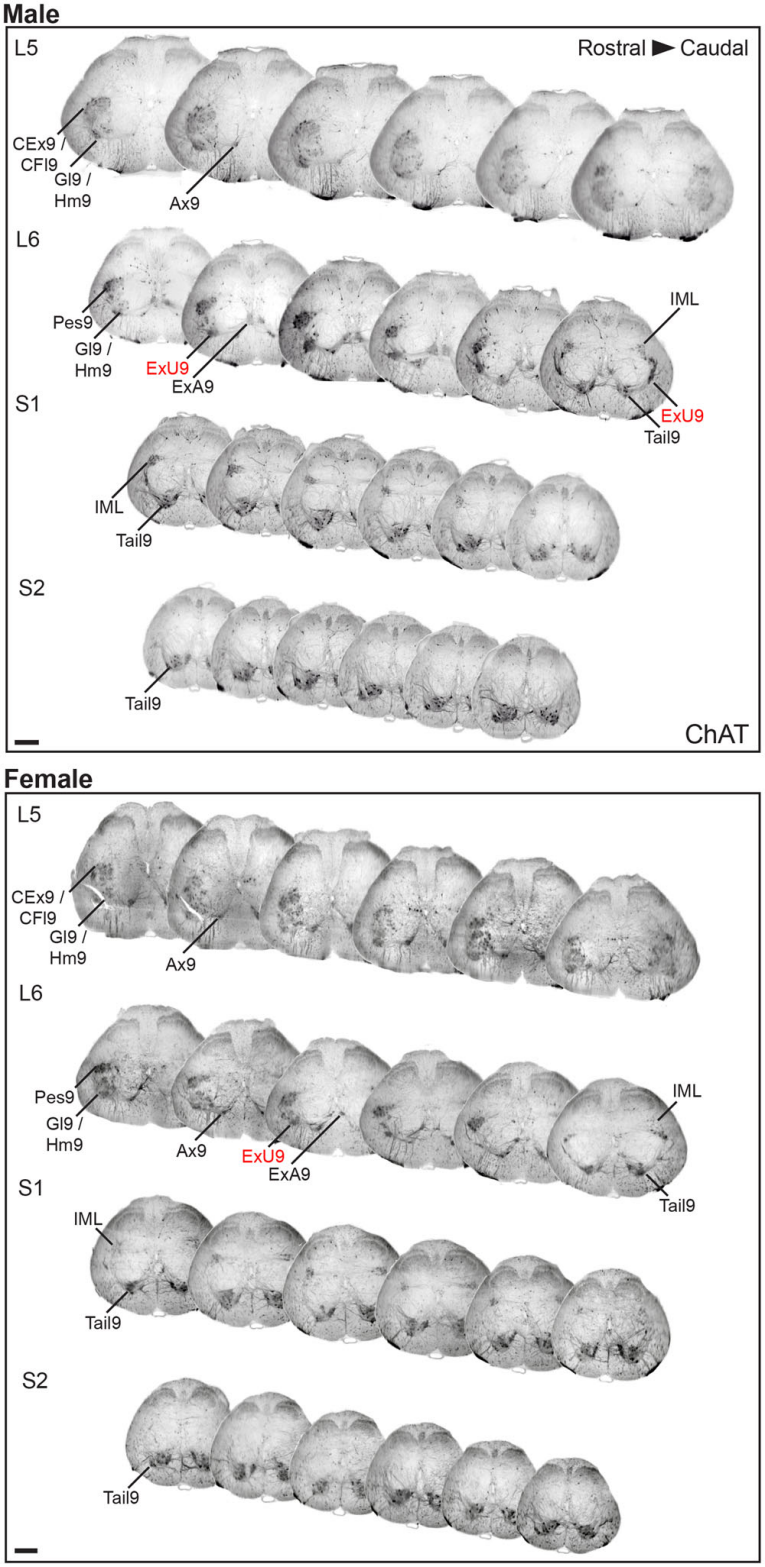
Comprehensive visualization of motor neuron topography in male and female rat lumbosacral spinal cord. Transverse virtual slices extracted at regular intervals across the 3D lumbosacral spinal cord dataset; six slices shown per segment. Spinal cord immunolabeled with choline acetyltransferase (ChAT). Motor columns labeled according to the rat spinal cord atlas (Watson et al. 2021). Labels in red indicate a correction compared to the Watson et al. (2021) rat spinal cord atlas based on the literature and our observations. Of all the lumbosacral motor nuclei, those innervating the urethral rhabdosphincter and ischiocavernosus (ExU9) and the anal rhabdosphincter and bulbocavernosus (ExA9) are consistently larger in males than female rats. CFl9, crural flexors; CEx9, crural extensors; Gl9, gluteal; Hm9, hamstring; Ax9, axial; Pes9, distal crural; IML, intermediolateral nucleus; Tail9, tail. Scale bar: 500 µm (applies to both panels).

**FIGURE 6:**
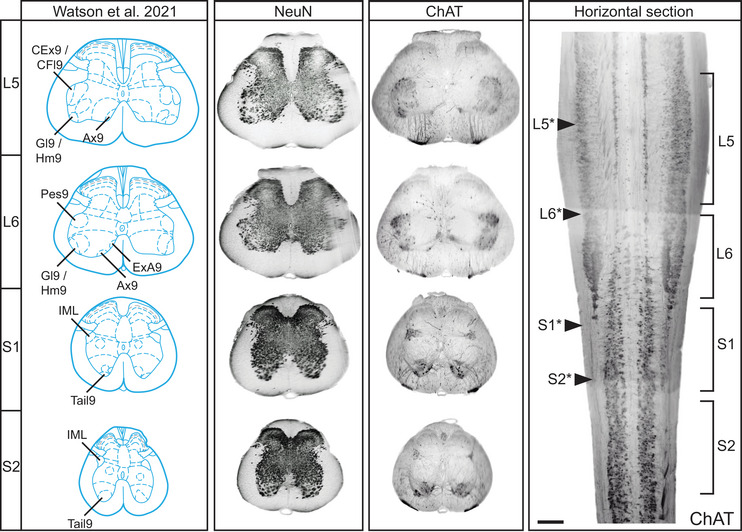
Identification of the rostrocaudal position of rat spinal cord atlas sections along the lumbosacral neuraxis. Matching representative diagrams of segments L5–S2 taken from the rat spinal cord atlas (Watson et al. 2021) to 40‐µm transverse virtual slices of cleared spinal cord labeled with either NeuN or choline acetyltransferase (ChAT) (male). The rostrocaudal position of each transverse virtual slice is indicated on a horizontal virtual slice (with arrows and asterisk) of the same spinal cord relative to the actual segment position, as determined by the ventral roots. Motor columns labeled according to the rat spinal cord atlas (Watson et al. 2021). CFl9, crural flexors; CEx9, crural extensors; Gl9, gluteal; Hm9, hamstring; Ax9, axial; Pes9, distal crural; ExA9, anal rhabdosphincter; IML, intermediolateral nucleus; Tail9, tail. ExU9 (urethral rhabdosphincter) is not indicated in L6 as this motor column is not present in such a rostral section and was misidentified in the rat spinal cord atlas as the motor column that is Ax9. Scale bar: horizontal section, 500 µm.

To account for strain differences and compare our 3D data to the 2D atlas maps from Wistar rats (C. Watson et al. 2009, 2021), we produced an ordered series of 24 virtual transverse slices (six per segment) of the L5–S2 segments in male and female spinal cords of Sprague–Dawley rats (*n* = 1 per sex). These were immunolabeled with ChAT to identify the MN nuclei and NeuN to label the soma of most spinal cord neurons, an approach widely used to reveal central nervous system cytoarchitecture. To provide a more granular resource for research on the lumbosacral cord, we next used these 3D datasets to produce an ordered series of transverse virtual slices of ChAT immunolabeling in male and female rat spinal cord (Figure 5). The most commonly used nomenclature collectively identifies ventral horn MNs as spinal cord lamina 9 (Rexed 1952, 1954), which is subdivided into nuclei identified by location (Barber et al. 1984; Swanson et al. 2024). However, the Wistar rat atlases introduce a new nomenclature that identifies the nuclei in spinal cord lamina 9 by the target of the largest MN pool found at that location. This nomenclature was used to annotate the atlas based on transverse virtual slices shown in Figure 5. This revealed an error in the spinal cord atlases of C. Watson et al. (2009, 2021), as the dorsomedial MN nucleus close to the midline (Figure 5) is identified as ExU9, which indicates it contains the MN pool for the external urethral sphincter. However, a comparative anatomical review in the same volume as the atlases shows the MN pools projecting to the urethral sphincter and ischiocavernosus muscles located in the lateral ventral horn (Holstege and Collewijn 2009). This is confirmed by multiple neural tracing studies in rodents that show these MN pools located in the dorsolateral MN nucleus on the lateral margin of the ventral horn (Schrøder 1980; McKenna and Nadelhaft 1986; Vizzard et al. 1995; Nadelhaft and Vera 1996, 2001; Karnup et al. 2024). Based on this evidence, we have annotated the DL nucleus as ExU9 in Figures 5 and 6.

The atlas of the adult rat spinal cord (C. Watson et al. 2009, 2021) provides a single map for each of the L5–S2 segments in the lumbosacral transition area (Figure 6, left panel). These show the IML (or SPSy) and eight lamina 9 nuclei, which are identified by a single muscle target of the motor pools located in each nucleus (Table 3). Using the series of virtual slices from our 3D male rat datasets, we identified the four virtual slices that most closely aligned with the atlas maps of the male spinal cord and show their actual locations on a horizontal view of the lumbosacral transition area (Figure 6, right panel). This revealed mismatches between our maps based on a 3D approach with segments defined by ventral roots (Sprague–Dawley rats) and the segmental representative 2D sections selected for the atlas (Wistar rats). First, our virtual slice resembling the 2D atlas view of L6 was located at the L5–L6 boundary. Even if there were no differences in rat strains, from our consistent findings across rats in this and a previous study (Fuller‐Jackson et al. 2021), a section at this level would not be expected to contain preganglionic neurons. Second, our virtual slice resembling the 2D atlas view of S2 was located within S1. Together, these comparisons demonstrated how the four 2D atlas maps of the lumbosacral transition area lack the spatial resolution needed to capture changes in the complex 3D topology of the MN pools that occur within the segments of the lumbosacral transition area.

**TABLE 3:** MN nuclei in atlases of the rat spinal cord (Watson et al. 2009, 2021).

MN nuclei	SMN target used for nomenclature
Ax9 Tail9	Axial m. Tail m.
Hm9 Gl9 CFl9 CEx9	Hamstring m. Gluteal m. Crural flexor m. Crural extensors
ExU9 Hm9 Gl9	External urethral sphincter Hamstring m. Gluteal m.
Pes9	Distal crural m.
ExA9	External anal sphincter

### 3.5 Dendritic Fields of Parasympathetic VMNs Reveal Sexual Dimorphism

Preganglionic VMNs have a characteristic multipolar morphology with long dendrites, which in a single neuron can span the distance from the midline of the spinal cord to the lateral edge of the white matter tracts (Nadelhaft and Booth 1984; Anderson et al. 2009; Peddie and Keast 2011). The relative density of somatic versus dendritic inputs has not been quantified, but large multipolar CNS neurons typically receive significantly more dendritic than somatic input (Nadelhaft and Booth 1984; Morgan et al. 1993; Morgan and Ohara 2001). This is consistent with projections of supraspinal inputs from the pontine micturition center to the soma of lumbosacral (parasympathetic) preganglionic MNs and following the medial dendrites out to the midline (Valentino et al. 1995, Valentino et al. 1999; Keller et al. 2018). This illustrates the important functional role of the dendritic field volume or “peri‐IML” in receiving and integrating spinal and supraspinal input.

**FIGURE 7:**
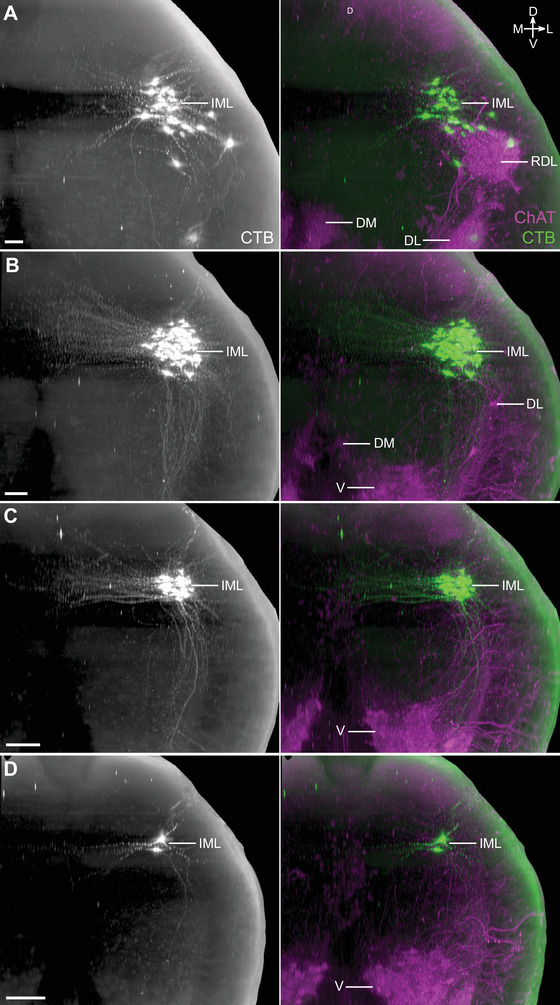
Dendritic field visualization achieved through retrograde filling of lumbosacral preganglionic neurons. Transverse virtual slices (100 µm) of cleared spinal cord from (A) caudal L6, (B) rostral S1, (C) middle S1, and (D) caudal S1. Spinal cord (female) immunolabeled for cholera toxin subunit B (CTB) and choline acetyltransferase (ChAT). IML, intermediolateral nucleus; RDL, retrodorsolateral motor column; DL, dorsolateral motor column; DM, dorsomedial motor column; V, ventral motor column. Scale bar: 100 µm.

**FIGURE 8:**
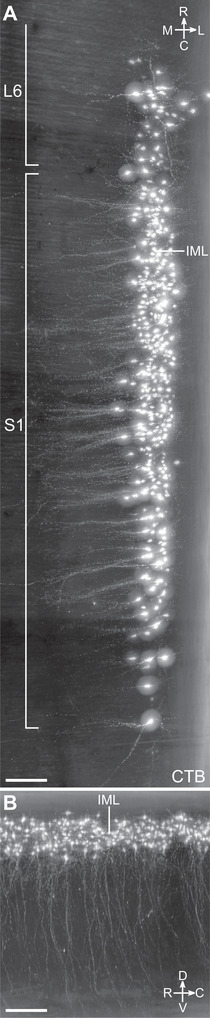
Horizontal and sagittal views of the dendritic field originating from sacral preganglionic neurons. Virtual slices (200‐µm thick) in (A) horizontal and (B) sagittal orientations, focusing on the intermediolateral nucleus (IML). Spinal cord (female) immunolabeled for cholera toxin subunit B (CTB). The intensity of the signal has been digitally increased to demonstrate dendrites in CTB^+^ neurons. Scale bar: 200 µm.

**FIGURE 9:**
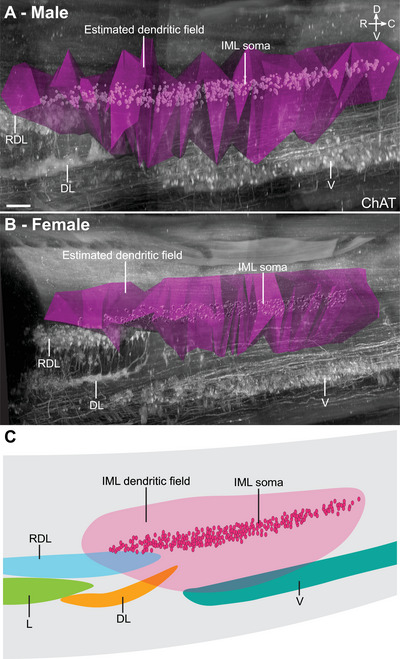
Estimated dendritic field representation of lumbosacral preganglionic neurons in the 3D context of the cleared lumbosacral spinal cord. Three‐dimensional volumes were generated according to the distribution of cholera toxin subunit B in the dendrites of preganglionic neurons located in the intermediolateral column of L6–S1 spinal cord in (A) male and (B) female rats. The estimated dendritic field volume was larger in males compared to female rats (see also Table 4). Spinal cord immunolabeled for choline acetyltransferase (ChAT). (C) Schematic representation of the dendritic field of lumbosacral preganglionic neurons in relation to nearby motor pools. RDL, retrodorsolateral motor column; DL, dorsolateral motor column; DM, dorsomedial motor column; V, ventral motor column. Scale bars: 100 µm (applies to both panels).

As CTB labeling identifies a much greater proportion of the MN dendritic tree than ChAT immunohistochemistry, we explored data from our MPG neural tracing experiments to assess how the dendrites of parasympathetic preganglionic neurons project in 3D space (Figures 7, 8, 9; Media 4 [https://doi.org/10.26188/25484332.v1]). Transverse views of the L6–S1 IML (Figure 7) showed how the dendrites have three preferred orientations: *medial*, often extending into the sacral dorsal commissural nucleus (SDCom) and close to the midline; *ventrolateral*, extending deep into the ventral horn; and *lateral*, extending into the surrounding spinal white matter tracts. These orientations are demonstrated in horizontal and sagittal views in Figure 8. Previous studies tracing single IML neurons in rodents and other species also report long rostrocaudal dendrites (Armstrong et al. 1983; Barber et al. 1984). These were difficult to view within the IML, where they run between the strongly fluorescent somata, but were shown to extend rostrally for some distance past the end of the preganglionic neuron column (Figure 8A).

The strong labeling of CTB^+^ lumbosacral (parasympathetic) preganglionic neurons encouraged us to visualize and quantify their dendritic field volume. The CTB immunofluorescence in dendrites was punctate, similar to that recently reported in central axons of sacral spinal sensory neurons (Fuller‐Jackson et al. 2021). We used the *Spots* function in Imaris to segment the fluorescent CTB^+^ puncta to obtain *XYZ* coordinates of a cloud of points outlining preganglionic neurons and their dendrites, which were then analyzed with a custom code that used a 3D composite convex hull to fit a surface around the point cloud (https://gitlab.unimelb.edu.au/lab‐keast‐osborne‐release/3d‐points‐volume‐estimation). A comparison of the total dendritic field volume of CTB^+^ lumbosacral preganglionic neurons in three male (Figure 9A) and female (Figure 9B) spinal cords identified a sex difference, being significantly larger in males (1.201 ± 0.188 mm^3^ vs. 0.624 ± 0.099 mm^3^; difference = 0.578 ± 0.219 mm^3^, two‐tailed *t‐*test: *p* = 0.0388, *df* = 5; Table 5).

**FIGURE 10:**
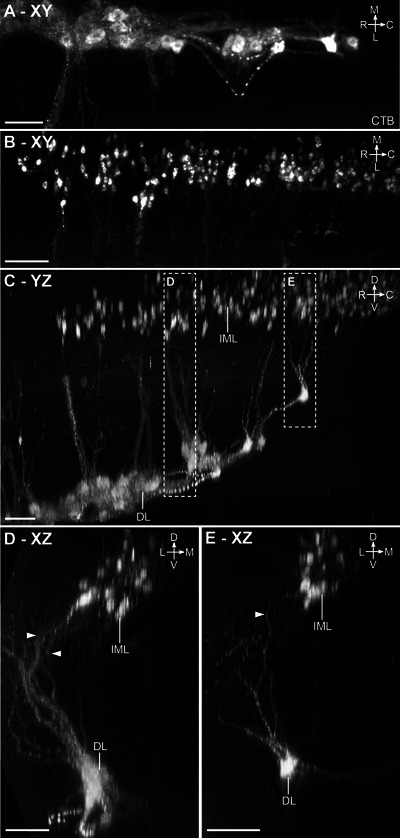
Close association between dendrites of lumbosacral visceral (preganglionic) and somatic motoneurons (MNs) identified with cholera toxin subunit B (CTB) retrograde filling. CTB was microinjected into the major pelvic ganglion. (A) Horizontal view of the CTB‐filled dorsolateral (DL) MNs and their dendritic bundles in cleared spinal cord (L6). (B) Horizontal view of the intermediolateral nucleus (IML) visceral MNs. (C) Sagittal view of DL MN dendrites projecting dorsally toward visceral MNs of the IML. (D, E) DL dendrites (indicated by arrows) in close proximity to less brightly labeled dendrites of the IML neurons, visualized in 100‐µm transverse virtual slices. *XY*, *YZ*, and *XZ* refer to the image orientation relative to the plane of image acquisition, with voxel size at 0.542 × 0.542 × 2.0 µm for *XYZ*. Scale bars: 100 µm (applies to all panels).

Many MNs in the spinal cord contribute to dendritic bundles. These are large groups of closely apposed parallel dendrites that can be electronically coupled by dendro‐dendritic gap junctions. It is hypothesized that dendritic bundles have a role in synchronizing activity within or across functional pools of MNs (Matthews et al. 1971; Personius et al. 2007; Bautista and Nagy 2014). The largest dendritic bundles are found between SMNs in the lumbosacral transition area (Roney et al. 1979; Schrøder 1980; Bellinger and Anderson 1987). To our knowledge, there are no reports of dendritic bundles including dendrites from both SMNs and preganglionic VMNs, but our analysis of dendritic field volumes showed that dendrites from these VMNs were close to the dorsolateral, retrodorsolateral, and ventral MN pools (Figure 9C). We further examined the spinal cords from the CTB injection study, where both lumbosacral preganglionic neurons and specific pools of SMNs were labeled from the MPG. Higher magnification images (Figure 10; Media 5 [https://doi.org/10.26188/25484764.v1]) showed there were many CTB^+^ dendrites in bundles linking the lumbosacral IML and ExU9.

### 3.6 Sexual Dimorphism of the Dorsolateral and Dorsomedial SMNs in L6

**FIGURE 11:**
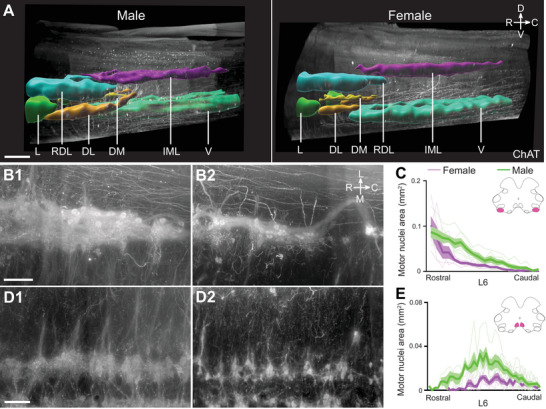
Sexual dimorphism of motor nuclei L6–S1. (A) Sagittal view of cleared lumbosacral spinal cord (L6–S1) from male and female rat, immunolabeled with choline acetyltransferase (ChAT) and motor nuclei segmented. In this 3D view, the dorsolateral motor column (DL) and dorsomedial motor column (DM) were larger in males. (B) Horizontal virtual slice of DL in male (B1) and female (B2) rats. (C) DL nuclei area from rostral to caudal L6 measured in transverse cryosections of male (*n* = 6) and female (*n* = 5) rats. (D) Horizontal virtual slice of DM in male (D1) and female (D2) rats. (E) DM nuclei area from rostral to caudal L6 measured in transverse cryosections of male (*n* = 6) and female (*n* = 6) rats. Note in B and D, the DL and DM nuclei appeared smaller in females, with a reduced density of dendritic bundles and projections. (C, E) Lines are mean, standard error of the mean, and individual traces contouring the relevant MN area on each section. L, lateral motor column; V, ventral motor column; RDL, retrodorsolateral motor column; IML, intermediolateral nucleus. Scale bars: 500 µm (A) and 100 µm (B, D).

Having identified a sex difference in the dendritic field volume of parasympathetic preganglionic neurons and establishing that they contribute to the same dendritic bundles as dorsolateral MNs, we next confirmed the sexual dimorphism of the dorsolateral (MNs of the urethral rhabdosphincter and ischiocavernosus) and dorsomedial (MNs of the anal rhabdosphincter pool and bulbocavernosus) motor pools. These nuclei are known to be sexually dimorphic, and their neurons are sensitive to androgens (Breedlove and Arnold 1981, 1980; Jordan et al. 1982; Sengelaub et al. 1989; Tobin and Payne 1991). In our 3D datasets, ChAT immunolabeling showed that the dorsolateral motor pool was larger and extended further caudally in males than in females (Figure 11A). However, the intensity of the ChAT fluorescence in the dendritic bundles that extend from MNs in the dorsolateral column made it difficult to count the less intensely labeled somata of the MNs. To address this, we reanalyzed an open dataset (Fuller‐Jackson et al. 2021) containing images of an ordered series of sections taken through the lumbosacral spinal cord in adult female (*n* = 6) and male (*n* = 6) Sprague–Dawley rats. This visualization of ChAT^+^ neurons in sections allowed us to segment individual MN somata from the raw signal. Plotting relative pool area along the rostrocaudal axis of the nucleus confirmed that the dorsolateral motor pool (Figure 11B,C) was larger and extended over a longer distance in males (dorsolateral transverse area: male = 36,627 ± 13,885 µm^2^, *n* = 6 vs. female = 21,128 ± 3895 µm^2^, *n* = 5; two‐tailed *t‐*test, *p* = 0.026, *df* = 10; Table 4). Similar analyses determined that the dorsomedial motor column (Figure 11D,E) was also larger in males (males, 16,242 ± 6040 µm^2^, *n* = 6 vs. females, 6191 ± 2486 µm^2^, *n* = 6; two‐tailed *t‐*test, *p* = 0.003, *df* = 11; Table 4).

**TABLE 4:** Counts of lumbosacral spinal cord VMNs (autonomic preganglionic MNs) and SMNs with projections to the major pelvic ganglia (MPG).

		1–L2						L6–S1						
		VMNs (Sympathetic)						VMNs (Parasympathetic)			SMNs			
		Total	IML			DCN		IML			DL	V (sacral)	V (lumbar)	
Sex	Subject^a^	CTB^+^	CTB^+^	ChAT^+^	%	CTB^+^	% total	CTB^+^	ChAT^+*^	%	CTB^+^	CTB^+^	CTB^+^	Sum
Female	731	137	23	522	4.4	114	83.2	593	652	91.0	1	33	0	34
	732	273	30	508	9.9	243	89.0	621	710	87.5	6	53	0	59
	733^b^	—	—	—	—	—	—	709	758	93.5	0	62	—	62
Male	714	224	78	778	10.0	146	65.2	723	903	80.1	81	24	32	137
	715	299	84	912	9.2	215	72.0	589	829	71.0	7	0	54	61
	716	367	107	957	11.1	262	71.0	694	801	86.6	71	2	23	96

*Note:* Projection neurons retrogradely labeled after bilateral CTB (cholera toxin beta subunit) injections into the MPG (major pelvic ganglia). Unilateral counts are shown, except for the midline DCN, which are bilateral across the entire nucleus.

Abbreviations: ChAT, choline acetyltransferase; DCN, dorsal commissural nucleus; DL, dorsolateral motor column; IML, intermediolateral nucleus; SMNs, somatic motor neurons; V, ventral motor column of either lumbar or sacral spinal cord; VMNs, visceral motor neurons.

^a^ Subject identifier in the open dataset (sparc.science).

^b^ Missing counts due to sample damage.

*Two‐tailed *t‐*test, females versus males, *p* = 0.033.

**TABLE 5:** Sex differences identified in the lumbosacral spinal cord of Sprague–Dawley rats.

Sex difference	Details	Statistical comparisons
Ventral (lumbar) SMN projections via the MPG	Only observed in males	—
Estimated dendritic field volume of L6–S1 IML neurons	Males; 1.201 ± 0.188 mm^3^, *n* = 3 Females; 0.624 ± 0.099 mm^3^, *n* = 3	Two‐tailed *t*‐test, *p* = 0.0388, *df* = 5
L6–S1 IML neuron counts	Males; 844 ± 30, *n* = 3 Females; 707 ± 31, *n* = 3	Two‐tailed t‐test, *p* = 0.033, *df* = 5
Dorsolateral motor pool area in transverse cryosections	Males; 36,627 ± 13,885 µm^2^, *n* = 6 Females; 21,128 ± 3895 µm^2^, *n* = 5	Two‐tailed *t‐*test, *p* = 0.026, *df* = 11
Dorsomedial motor pool area in transverse cryosections	Males; 16,242 ± 6040 µm^2^, *n* = 6 Females; 6191 ± 2486 µm^2^, *n* = 6	Two‐tailed *t‐*test, *p* = 0.003, *df* = 11

### 3.7 Distinctive Cellular and Subcellular Features of MN Pools in the Lumbosacral Visceral–Somatic Transition Zone

**FIGURE 12:**
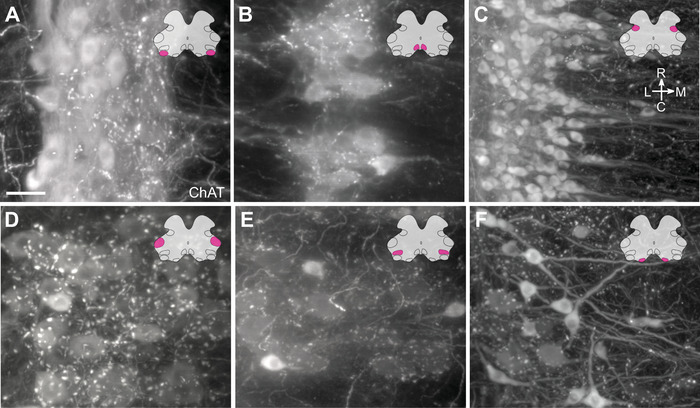
Motor neuron morphology in the lumbosacral spinal cord. Horizontal virtual slices of (A) dorsolateral (DL), (B) dorsomedial (DM), (C) parasympathetic preganglionic (IML), (D) retrodorsolateral (RDL), (E) lateral (L), and (F) ventral (V) motor columns in the cleared lumbosacral spinal cord (L6–S1, male rat). Spinal cord immunolabeled for choline acetyltransferase (ChAT). Scale bar: 50 µm.

**FIGURE 13:**
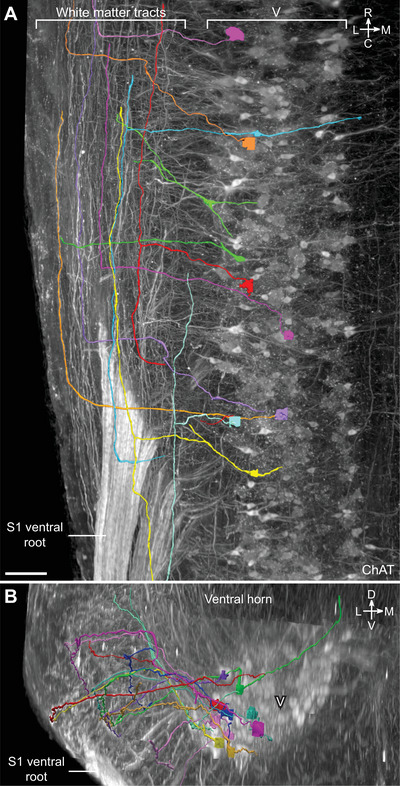
Sacral somatic motor neurons (SMNs) possess intersegmental axonal projections. (A) Horizontal view of the levator ani and tail SMNs in the ventral motor column (V) and proximal white matter tracts in cleared S1 spinal cord (male) with individual MN axons traced and indicated by different colors. (B) Zoomed‐in horizontal view highlighting an SMN axon projecting into the white matter tracts. (C) Transverse view of the ventral horn of the same cleared S1 spinal cord. ChAT, choline acetyltransferase. Scale bars: 100 µm.

Light sheet microscopy provided a unique opportunity to build a true 3D map of the lumbosacral MN pools and establish their mesoscale and macroscale relationships. These large 3D datasets were also of sufficient resolution to identify and compare specific microscale (cellular and subcellular) features of each MN pool, including aspects of their somata, proximal dendrites, and axon projections. Here, we have aggregated our descriptive findings for each of the MN pools, integrating observations illustrated in several previous figures and extended in Figures 12 and 13 and Media (Media 4 [https://doi.org/10.26188/25484332.v1], Media 5 [https://doi.org/10.26188/25484764.v1], and Media 6 [https://doi.org/10.26188/25484788.v1]. Together, these revealed differences across groups, in agreement with previous observations from histological sections, but also identifying novel features. Observations were gathered from all cleared ChAT^+^ spinal cords (*n* = 8 male, *n* = 4 female), and representative images were generated from a single male rat spinal cord (Figures 12 and 13). These have been grouped according to the MN pool.

#### 3.7.1 Parasympathetic (Preganglionic) VMNs

The most rostral neurons in the parasympathetic IML appeared dorsomedial to the retrodorsolateral MN pool in mid‐L6 (Figures 4 and 9). Across the caudal sections of L6, the IML increased in size and neuron number, taking up the entirety of the intermediolateral region in lamina 5/6. This column of VMNs continued through S1 but did not extend into the S2 segment. At higher magnification, the VMNs formed a dense column of predominantly spindle‐shaped multipolar neurons, which had smaller somata and were less rounded than SMNs (Figure 12C). Dendrites were only partly revealed by ChAT immunofluorescence but could be seen extending medially toward the central canal and running longitudinally between somata in the IML column; these were revealed more clearly by the retrograde tracer, CTB (Figures 7, 8, 9; Media 4 [https://doi.org/10.26188/25484332.v1]). Proximal dendrites extending laterally into the white matter tracts were also clearly detectable with CTB but not ChAT. No large ChAT^+^ C‐boutons, characteristic of typical alpha MNs, were associated with the IML.

#### 3.7.2 Urethral Rhabdosphincter and Ischiocavernosus (Dorsolateral)

The dorsolateral motor column comprises the motor nucleus innervating the urethral rhabdosphincter and ischiocavernosus muscle (Table 3), which first appears adjacent to the ventrolateral edge of the lateral motor column; this corresponds to the second digital slice of L6 (Figure 6). By the caudal end of L6, the dorsolateral motor column had reduced in size to only one to three neurons per nucleus per slice. Proceeding rostrocaudally through the dorsolateral column, the MNs gradually shifted dorsally to occupy the most lateral edge of the ventral horn and progressively showed a higher density of dorsally orientated dendritic bundles. Although the somata of MNs in the dorsolateral column did not extend to S1, their dendritic bundles were visible in the rostral slice of S1. It is important to note that without muscle‐specific retrograde tracing (McKenna and Nadelhaft 1986) (Table 2), urethral rhabdosphincter MNs cannot be distinguished from ischiocavernosus MNs. Therefore, in the following morphological descriptions, we cannot define which features apply to each functional class. Dendritic bundles of dorsolateral neurons extended rostrocaudally, as well as laterally and dorsally along the ventral boundary of the gray matter, with no major bundles extending dorsomedially into the deeper gray matter regions, such as toward lamina 10 (Media 5 [https://doi.org/10.26188/25484764.v1]). Higher magnification views showed the heterogeneity across the dorsolateral MN pool in the distribution of C‐boutons associated with somata and the density of dendritic bundles surrounding different regions of the dorsolateral column, with some dorsolateral neurons having a dense supply and others having none (Figure 12A).

#### 3.7.3 Anal Rhabdosphincter and Bulbocavernosus (Dorsomedial)

SMNs of the dorsomedial column supply the anal rhabdosphincter and bulbocavernosus muscles (Table 3). The dorsomedial motor column is also referred to as the spinal nucleus of the bulbocavernosus (Sengelaub and Forger 2008). As with the dorsolateral column, these two intermingled groups of neurons cannot be distinguished using ChAT immunolabeling alone. In rostral L6, the dorsomedial MNs appeared in the most medial portion of the ventral horn, immediately ventral to the central canal and lamina 10 (Figure 6). The dorsomedial MN pool increased in size from rostral L6 until the midpoint, after which the nuclei decreased in size until rostral S1, wherein only the ChAT^+^ dendrites of the motoneurons were visible (digital slices three and four of L6; Figure 6). Inspection at higher magnification demonstrated the dense meshwork of dendritic bundles surrounding the dorsomedial MNs (Figure 12B), although this was less dense than in the dorsolateral motor column. These bundles projected laterally in strikingly regular intervals across the rostrocaudal neuraxis (Figure 11D).

#### 3.7.4 Lower Limb (Retrodorsolateral and Lateral)

In rostral L6, three MN nuclei innervate muscles of the lower limb; retrodorsolateral MNs project to the distal crural muscle, and MNs of the lateral motor column project to the gluteal and hamstring muscles (Table 3). Located in the dorsolateral corners of the ventral horn, the retrodorsolateral motor column was a compact aggregate of predominantly large stellate and medium‐sized round MNs (Figures 6 and 12D). Small, intensely labeled spindle‐shaped neurons were also visible (Figure 12D). There were no visible bundles of dendrites; however, C‐boutons were present around many large‐ and medium‐sized MNs (Figure 12D). By caudal L6, the retrodorsolateral pool was much smaller, with few MNs remaining and none appearing in S1 (Figure 6). In the ventrolateral corner of the ventral horn in rostral L6, lateral MNs also comprised large, medium, and small MNs of stellate, round, and spinal morphology, respectively (Figure 12E). Compared to MNs of the retrodorsolateral region, lateral MNs were less densely packed (Figures 6 and 12D). Halfway through L6, the lateral motor column ended, to be replaced completely by dorsolateral MNs (the fourth slice of L6; Figure 6).

#### 3.7.5 Axial (Ventral)

Ventral MNs of the lumbar cord project to the axial muscles and are located in the most ventromedial aspect of the L5 ventral horn (Figure 6). In L6, this is a small nucleus, with only one or two neurons per slice observed in rostral L6 and ending by the third slice (Figure 6). At this point, it was replaced by the ventral motor column of the sacral spinal cord (see below).

#### 3.7.6 Levator Ani and Tail (Ventral)

The levator ani and tail muscles are innervated by the ventral motor pool, which appeared in the middle of L6 in the position previously occupied by axial‐innervating ventral MNs in the more rostral slices (third slice of L6; Figure 6). In the fourth slice, the size of the ventral MN column greatly increased, and by the most caudal slice of L6, the column was expanded in size such that throughout the S1 and S2 segments, it occupied around half of the ventral horn. At higher magnification, ventral MNs in the ventral horn of the sacral spinal cord exhibited the greatest disparity between neuronal subtypes (Figure 12F). Large stellate motoneurons with weak ChAT immunolabeling were surrounded by the greatest density of C‐boutons. Medium‐sized round MNs had stronger ChAT immunolabeling but fewer C‐boutons around their soma. Small spindle‐shaped MNs devoid of C‐bouton contacts were the most intensely immunolabeled MNs.

Of the lumbosacral motor nuclei inspected at higher magnification, the axons of the ventral MNs were the most strongly immunolabeled with ChAT. Many could be followed into the white matter tracts, turning 90° to project rostrally amid a column of ChAT^+^ axons in the ventrolateral white matter tract (Figure 13; Media 6 [https://doi.org/10.26188/25484788.v1]). Tracing these axons in Neurolucida 360 showed that all axons travelling via the white matter reached the L6–S1 boundary, presumably continuing to rostral segments. MNs that possessed these axonal projections into the white matter were of no single type, that is, including both small spindle‐shaped neurons and large neurons surrounded by C‐boutons. Some axons instead turned to project caudally; however, this was less frequently observed. In two cases, axons were found to bifurcate in the white matter, projecting in both directions of the neuraxis (Figure 13A).

### 3.8 A Comprehensive Map of Lumbosacral Motor Innervation

**FIGURE 14:**
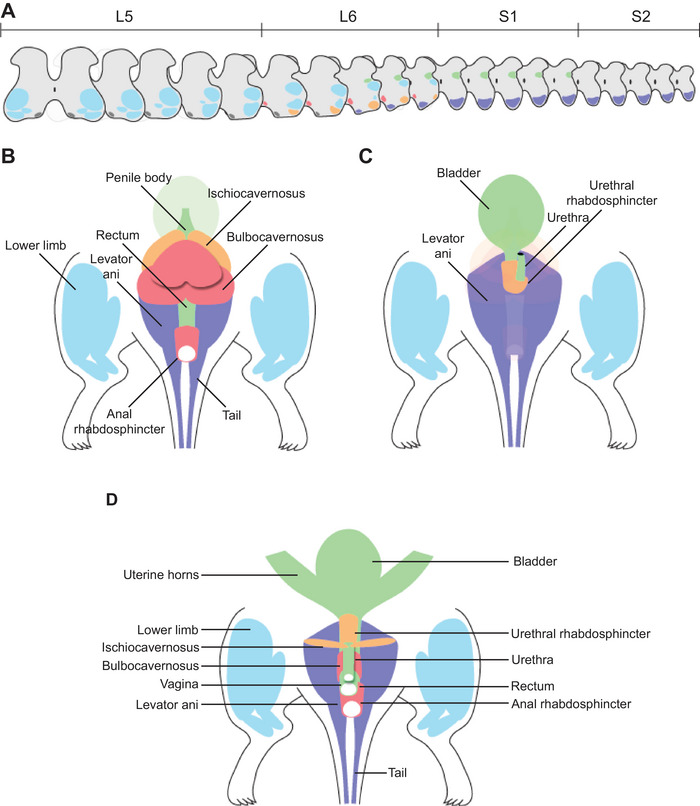
Graphical summary of lumbosacral motor nuclei topography and innervation. (A) Topography of motor nuclei in the transverse L5–S2 spinal cord. Colorization of motor nuclei matches that of the muscles in B–D, which illustrate the muscles and organs innervated by the somatic and visceral components of the pelvic motor system. (B, C) Male rat from dorsal (B) and ventral (C) views. (D) Female rat, ventral view.

The topographical organization of the SMN and VMN nuclei in the lumbosacral cord, particularly the complexity of the L6 segment, requires a more comprehensive graphical representation than is currently available. Based on our multiscale mapping, we created a series of schematics indicating the approximate position of each of the SMN and VMN nuclei as they would appear in transverse sections (Figure 14A). These have been colored according to the somatic muscle or pelvic organ that they regulate, in both male (Figure 14B,C) and female (Figure 14D) Sprague–Dawley rats.

## 4 Discussion

We applied advanced whole‐mount immunofluorescence 3D microscopy (Belle et al. 2017; Fuller‐Jackson et al. 2021; Blain et al. 2023) to study the pelvic MN system in female and male rats. This highly specialized motor circuit, formed by autonomic preganglionic VMNs and SMNs, controls motor activity during excretory, sexual, and reproductive behaviors (Anderson et al. 2009; Holstege and Collewijn 2009; de Groat 2018). Unusual features include extensive morphological sex differences, motor patterns that combine visceral and somatic activity, and psychogenic activation during higher order behaviors (Breedlove and Arnold 1981; Thor and de Groat 2010; Hou et al. 2016; Keller et al. 2018). Three‐dimensional analysis of spinal cord neuroanatomy has been limited by the technical challenges of producing reconstructions from sections, but with new technologies, we performed the first multiscale 3D study integrating macroscopic organization and microscopic morphology of pelvic MNs. This revealed SMN projections via the MPG, the relationship between dendrites of pelvic SMNs and VMNs, intersegmental axons arising from sacral SMNs, and sex differences in dendritic field volume of sacral VMNs. Our data will be available on an open science platform (sparc.science) to allow further exploration.

We first revealed the macroscopic organization of MNs using the characteristic size, morphology, and location of cholinergic MNs to distinguish them from cholinergic interneurons dispersed in other spinal regions (Barber et al. 1984). During development, MNs segregate into longitudinal medial and lateral columns (Dasen 2022), which postnatally are interrupted by the enlargements to produce subcolumns of morphologically distinct MNs (Barber et al. 1984). This 3D organization was shown by segmenting these columns and projecting digital 3D models onto raw images. This demonstrated how the composition of the functionally distinct MN columns differs in the upper, middle, and lower segments of the lumbosacral cord. The lateral SMN columns targeting the hindlimbs occupy the middle region at the center of the enlargement (Nicolopoulos‐Stournaras and Iles 1983). These extend rostrally to overlap with the ventral SMN columns and the sympathetic VMN columns (Anderson et al. 2009; Scott‐Solomon et al. 2021; Qi et al. 2022) and extend caudally where the lateral column overlaps with a single parasympathetic VMN column and four SMN columns (Schrøder 1980; Barber et al. 1984). The increased number and neuroanatomical complexity of these MN columns correspond with this region being the major source of motor output to the pelvis and urogenital region (Holstege and Collewijn 2009; de Groat 2018).

We identified both sympathetic (L1–L2) and parasympathetic (L6–S1) pelvic VMNs by neural tracing from their primary output target, the MPGs (Hancock and Peveto 1979a, 1979b; Watkins and Keast 1999). This confirmed previous evidence indicating major differences between pelvic VMNs and other thoracolumbar VMNs. First, major targets of the thoracolumbar (sympathetic) IML are the paravertebral (sympathetic chain) ganglia that control cardiovascular (vasoconstrictor) function and thermoregulation (Anderson et al. 2009). We confirmed that most thoracolumbar VMNs regulating pelvic organs are instead located in the DCN, a midline column (Hancock and Peveto 1979a, 1979b; Watkins and Keast 1999). Second, parasympathetic VMNs located in the L6–S1 IML target only the MPG, that is, exclusively regulate visceral targets, again contrasting with a major function of the sympathetic IML. These functional differences between thoracolumbar IML, DCN, and lumbosacral IML are relevant to recent transcriptomic classification (Blum and Gitler 2022). Functional specialization of the VMNs in IML above and below the lumbar enlargement correlates with their distinct transcriptomes (Alkaslasi et al. 2021; Blum et al. 2021; Liau et al. 2023). VMNs in the DCN that preferentially output to pelvic visceral motor targets have not yet undergone this analysis, which is predicted to reveal neural classes different from the IML in the thoracolumbar spinal cord.

Retrograde labeling of some SMNs in dorsolateral and ventral MN pools (both upper lumbar and sacral) by MPG microinjection of CTB indicates that specific SMN groups innervate their target muscles via an MPG trajectory, which has potential implications for surgical procedures and therapeutic neuromodulation. In cats and humans, there are multiple reports of communicating branches between the pudendal nerve or sacral spinal roots and the pelvic ganglia/pelvic nerve (Langley and Anderson 1895; Drizenko et al. 2003; Mauroy et al. 2003; Alsaid et al. 2011; Bertrand et al. 2016). Our result is also consistent with studies in female rats, where transection of the pudendal nerve motor branch innervating the urethral rhabdosphincter failed to abolish the vaginocervical reflex, while a proportion of male rats slowly recovered urinary continence (Juárez et al. 2012).

To demonstrate the benefit of the 3D approach for anatomical mapping, we produced a series of virtual transverse slices (six per segment), immunolabeled for ChAT and the cytoarchitectural marker, NeuN. This revealed a limitation of the current atlases that select a single location to represent each segment, especially for L6, where there is a major change along its length (C. Watson et al. 2009, 2021). Specifically, the IML extends caudally from mid‐L6 yet in the atlases is shown as absent in L6. We also note that the atlases show the IML extending to S2, whereas we rarely find IML in this segment. This discrepancy could be due to such an unusual case in which a rostral section of S2 was selected for the atlas, or it could reflect a strain difference in the rostrocaudal location of the IML in Sprague–Dawley rats (this study) versus Wistar rats (atlases) (Pascual et al. 1989). The complex 3D topology of MN columns with segments revealed a limitation of the available 2D atlases of the rat spinal cord, which only provide a single transverse map of each segment and do not show the IML in segment L6 (C. Watson et al. 2009, 2021)(Table 3). However, the spinal cord atlases are based on Wistar rats, and the absence of the IML in L6 could be due to strain differences, as in the rostrocaudal location of the parasympathetic preganglionic neurons (Pascual et al. 1989; L6–S1 in Sprague–Dawley, S1–S2 in Wistar).

Recent atlases (C. Watson et al. 2009, 2021) have revised the ontological nomenclature used to identify SMN regions (Rexed 1952, 1954; Barber et al. 1984; Molander et al. 1984; Swanson et al. 2024) by naming nuclei in lamina 9 after the dominant MN pool in the corresponding location. The SMN pools in the pelvic motor system have been identified by neural tracing and functional experiments (Schrøder 1980; Thor and de Groat 2010). Reviewing these reports identified an anomaly in the rodent atlases (C. Watson et al. 2009, 2021), as the dorsolateral nucleus, referred to as ExU9, that targets the urethral rhabdosphincter (and ischiocavernosus muscle in males) is shown in the ventromedial column and not the ventrolateral column—the latter being the correct location, supported by strong functional anatomical evidence from multiple studies (Schrøder 1980; McKenna and Nadelhaft 1986; Vizzard et al. 1995; Nadelhaft and Vera 1996, 2001; Karnup et al. 2024). The revised nomenclature is simple and clear but also can be misleading, as other SMN pools can be co‐located or split across regions in different columns (Schrøder 1980) (Table 2). The nomenclature of ExU9 and ExA9 usefully distinguishes their sphincter targets, but in male rats, the dominant SMN population targets the ischiocavernosus and bulbocavernosus muscles, respectively (McKenna and Nadelhaft 1986). Further examination of these mixed neuron populations may identify unique molecular profiles to enhance future mapping.

Spinal cord MNs are typically multipolar with long dendrites extending through a large volume of gray and white matter (Vrieseling and Arber 2006; Fukuda et al. 2020). In most CNS neurons, dendrites receive significantly greater input than the soma; therefore, the perinuclear area defined by dendritic fields is important for understanding circuit function. We found that the perinuclear volume of the L6–S1 IML was larger in males than in females. To our knowledge, this is the first report of sexual dimorphism of the VMN dendritic field. We also identified a greater number of VMNs in the lumbosacral IML of male rats; this sex difference was not reported by an earlier study (Nadelhaft and Booth 1984); however, this study used different approaches (retrograde tracing and sections). A technical limitation of our study was that the group sizes were too small to support analysis of the effects of the estrous cycle in female rats. In male rats, estrogen produced by aromatization of testosterone maintains the dendritic morphology of SMNs innervating the pubococcygeus muscle, and in castrated rats, estradiol restores normal dendritic morphology after 2 weeks (Manzo et al. 1999). Phasic remodeling of lumbosacral VMNs or SMNS caused by fluctuating steroid levels could increase experimental variance and mask other sexually dimorphic features of these neurons.

Together, our data (Table 4) add to the extensive evidence of sexual dimorphism in the pelvic motor system (Jordan et al. 1982; Anderson et al. 2009; Forger 2009; Thor and de Groat 2010; Oti and Sakamoto 2023). These differences are important for studies of spinal cord injury and neurodegenerative conditions.

We identified a prominent dendritic bundle connecting the L6–S1 IML and the dorsolateral motor column. Dendritic bundles develop postnatally (Bellinger and Anderson 1987; Markham et al. 1991), and in rats, the largest are formed by SMNs in the lumbosacral spinal cord, with the ventrolateral bundle containing 1200–1600 dendrites (Roney et al. 1979). VMNs in the L1–L2 and L6–S1 IML also form prominent, evenly spaced, long dendritic bundles that extend medially to appear ladder‐like in horizontal sections (Peddie and Keast 2011). These bundles have only been examined in transverse or horizontal sections (Roney et al. 1979), but we found that some CTB^+^ dendrites of L6–S1 VMNs were oriented rostrodorsally and joined dendritic bundles formed by the dorsolateral SMNs. A major output of the sacral IML is bladder contraction that is coordinated with inhibition of the urethral rhabdosphincter. Therefore, the colocation of visceral and somatic dendritic bundles could have functional significance. For example, there is evidence suggesting that electrical coupling of bundled dendrites synchronizes SMN activity and their output to functionally coupled groups of striated muscles (Roney et al. 1979; Personius et al. 2007).

We focused on the pelvic motor system and could only partly explore the neuroanatomical detail available in these rich datasets. This was highlighted by the resolution of the raw 3D images, revealing single axons projecting from sacral SMNs to enter the white matter and then abruptly turn to run for long distances in white matter tracts. This could correspond to sacral MN pools that have ascending intersegmental inputs to drive activity in lumbar locomotor pattern generators (Cazalets and Bertrand 2000; Sourioux et al. 2018; Mille et al. 2021).

This study has used whole‐mount immunostaining and advanced 3D microscopy to produce a 3D atlas of the pelvic MN system in the caudal spinal cord of adult female and male rats. The datasets are also available as an open resource (sparc.science) to support 3D visualization and further analysis of VMNs and SMNs identified by ChAT immunolabeling. This is the first multiscale analysis of the specialized caudal motor system that controls the sexually dimorphic genitourinary organs and specialized striated muscles in the pelvis. However, further study is needed to determine how the spinal neural circuit is organized and functions in directing reflexogenic and psychogenic (conscious) visceral and somatic motor activity during urinary continence and voiding, scent marking (in some species), defecation, reproduction, and sexual activity.

## Author Contributions

J.‐P.F.‐J. performed experiments, analyzed and interpreted data, and contributed to the writing of the manuscript. Z.Y. analyzed data and wrote code. N.M.W., A.W., and N.E.C.J. performed experiments. J.R.K. and P.B.O. conceptualized the project, designed experiments, interpreted data, and contributed to the writing of the manuscript.

## Conflicts of Interest

The authors declare no conflicts of interest.

## Peer Review

The peer review history for this article is available at https://doi.org/10.1002/cne.70109.

## Data Availability

Raw data from this study will be published under an open‐access license on https://sparc.science/.
